# Species-dependent protoplast enlargement involves different types of vacuole generation in bacteria

**DOI:** 10.1038/s41598-020-65759-7

**Published:** 2020-06-01

**Authors:** Sawako Takahashi, Marin Mizuma, Satoshi Kami, Hiromi Nishida

**Affiliations:** Research Center and Department of Biotechnology, Toyama Prefectural University, 5180 Kurokawa, Imizu, Toyama, 939-0398 Japan

**Keywords:** Membrane biophysics, Vacuole

## Abstract

Vacuole generation occurs frequently during the enlargement of bacterial protoplasts and spheroplasts. Gram-positive *Enterococcus faecalis* protoplasts and gram-negative *Lelliottia amnigena* spheroplasts had large and small vacuoles inside the cytoplasm, respectively. Although no vacuoles were found at the early stage of cell enlargement, all enlarged cells used in the microinjection procedures had vacuoles. The plasma membrane of *L. amnigena* was more flexible than that of *E. faecalis*. In addition, *E. faecalis* protoplasts had unique discoidal structures as well as spherical structures in the cytoplasm. Our findings showed that the number of vacuoles increased as the *L. amnigena* plasma membrane expanded and that the size of vacuoles increased as the *E. faecalis* plasma membrane expanded, suggesting that bacterial cell enlargement involved vacuole generation. Thus, biosynthesis of the plasma and vacuolar membranes was synchronous with the bacterial cell enlargement. Differences in the plasma membrane flexibility might influence the different types of vacuole generation.

## Introduction

The aim goal of our research was to generate a novel organism with a defined genome. We enlarged different bacterial cells for microinjection of various substances, for example, heterogeneous or designed genomic DNA. In order to generate the enlarged cells suitable for microinjection, it was essential to regulate cell enlargement and maintain the enlarged size of the cells. Although bacterial cells cannot enlarge due to the presence of cell wall (peptidoglycan), the spheroplasts or protoplasts could enlarge under suitable conditions^1–8^. In this study, the cells lacking peptidoglycan with an outer membrane are called spheroplasts, and those without an outer membrane are called protoplasts. Generally, bacterial cells are only a few micrometres in diameter and do not contain a vacuole. However, all enlarged cells used in the microinjection procedures had vacuoles. Thus, to achieve microinjection of various substances directly into the cell cytoplasm it is necessary to avoid microinjections into the vacuoles. We had a question whether vacuole generation can be regulated or not in the cell enlargement process. In this study, we investigated whether the bacterial cell enlargement involved vacuole generation or not.

Electron microscope observations revealed that the vacuoles in the *E. coli* and *B. subtilis* protoplasts were surrounded by single membranes, which did not contain DNA^2,3^. These vacuoles could be isolated from the protoplasts by the removal of the plasma membrane^2,3^. This suggested that the mature vacuoles were not connected to the plasma membrane. The vacuolar membrane of the *E. coli* protoplasts had the plasma membrane proteins, but outer membrane proteins were not detected^2^. In addition, the respiratory chain and F_0_F_1_-ATPase were detected in the vacuolar membrane of the *E. coli* protoplast^2^ and the respiratory chain was also detected in the *B. subtilis* protoplast^3^. These results showed that the vacuolar membrane components were same or very similar to the components of the plasma membrane. Furthermore, patch clamp analyses showed that the *E. coli* and *B. subtilis* vacuoles have everted membrane^2,3^. These results strongly suggested that the vacuolar membrane was generated by endocytosis of the plasma membrane^2,3^. Thus, it can be concluded that the vacuoles were generated from the plasma membrane and the vacuolar membrane was connected to the plasma membrane at the early stage of cell enlargement, following which the vacuoles grew independently of the plasma membrane.

In our laboratory, we incubated and enlarged bacterial protoplasts and spheroplasts in Difco Marine Broth 2216 (DMB) or modified DMB containing penicillin^4–8^. DMB contains four major metal salts, CaCl_2_, KCl, MgCl_2_, and NaCl. Thorough investigation of the compositions of medium metal salts showed that the presence of Ca^2+^ or Mg^2+^ required for spheroplast enlargement in *D. grandis*^7^. This finding revealed that metal ions play an important role in the enlargement. Furthermore, the total lipid composition changed during the enlargement of the *D. grandis* spheroplasts^7^. During the *D. grandis* cell enlargement, the outer membrane expanded much faster than the inner membrane^7^. In order to examine the effects of metal salts on the plasma membrane expansion and vacuole generation, the enlargement was performed under 16 different conditions, based on the reported effects of metal salts on the enlargement of *D. grandis* spheroplasts^7^ (Supplementary Table 1).

Among the enlarged bacterial cells in our laboratory, *L. amnigena* spheroplasts and *E. faecalis* protoplasts enlarged to a micro-injectable size (>20 µm). *L. amnigena* has the largest number of vacuoles, and *E. faecalis* has the largest vacuoles in the cytoplasm. In this study, we compared vacuole formation and membrane property of *L. amnigena* and *E. faecalis* enlarged cells. We studied the *E. faecalis* and *L. amnigena* enlarged cells by using optical and electron microscopy and examined the membrane property by microinjection.

*Lelliottia amnigena*, which belongs to the Enterobacteriaceae family, is a gram-negative, rod-shaped bacterium^9^ (Supplementary Fig. 1a). Enlarged spheroplasts of *L. amnigena* have outer and plasma membranes and generate a significant number of vacuoles in the cytoplasm (Supplementary Figs. 1b and 2a). The rate of expansion and synthesis of the outer membrane is higher than that of the plasma membrane, and consequently a large periplasmic space is formed^4,10^. A fluorescent image revealed that the vacuole and the large periplasmic space were not stained by the DNA-staining reagent 4′,6-diamidino-2-phenylindole (DAPI)^10^.

*Enterococcus faecalis*, which belongs to the Enterococcaceae family, is a gram-positive, spherical-shaped lactic acid bacterium^11^ (Supplementary Fig. 1c). During the enlargement of their protoplasts, the vacuole proceeds to press the plasma membrane, and finally the cell cannot maintain its spherical shape; this phenomenon has not been observed in other bacteria. Novobiocin^12^, a DNA replication inhibitor, inhibits the plasma membrane expansion and generation of vacuoles, indicating that DNA replication is associated with the protoplast expansion in *E. faecalis*^8^. In addition, the removal of novobiocin from the incubation medium leads to the plasma membrane re-expansion and re-generation of the vacuoles^8^. Thus, in the process of the *E. faecalis* protoplast enlargement, the expansion of plasma membrane was associated with vacuole generation.

These findings strongly suggest that the biosynthesis of the plasma and vacuolar membranes occurred simultaneously during the protoplast or spheroplast expansion. Here, in order to elucidate this process, we observed and compared the enlarged protoplasts and spheroplasts of *E. faecalis* and *L. amnigena*.

## Results

### Effect of metal salt composition on plasma membrane expansion and vacuole generation of *E. faecalis* and *L. amnigena*

**Figure 1:**
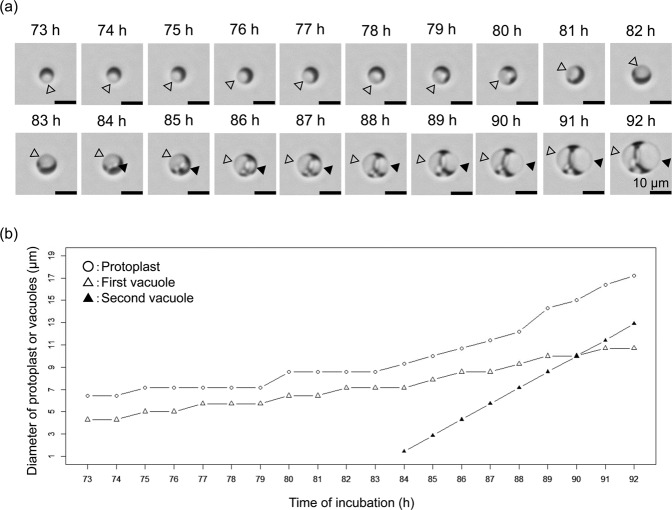
Time-lapse observation of *E. faecalis* protoplast enlargement. The protoplast after 73 h of incubation in DMB containing penicillin G was used. (**a**) Bright field microscopy images of time-lapse observation. White and black arrowheads indicate the first (previously formed) and second vacuoles, respectively. (**b**) Relation between the time of incubation and the diameter of protoplast or vacuoles. Bright field microscopy images were captured using a Keyence BZ-X710 microscope. Scale bar = 10 µm.

Among the 16 different combination of metal salts, the largest protoplasts of *E. faecalis* were found in DMB (Supplementary Fig. 3). The presence of Ca^2+^, K^+^, or Na^+^ enhanced the protoplast enlargement (Supplementary Figs. 3, 4). In DMB, the plasma membrane expanded with vacuole generation in the cytoplasm^8^ (Fig. 1). The time-lapse microscope observation revealed that the expansion rate of the small vacuole (the second vacuole) was faster than that of the senior vacuole (the first vacuole) and they eventually became the same size (Fig. 1).

In *L. amnigena*, the plasma membrane expansion was activated in the modified Marine Broth MMB3CaKMg, which led to inhibit a large periplasm generation (Supplementary Figs. 2b and 5). Surprisingly, the rate of enlargement was very rapid, and the collapse of the enlarged cell was also fast (Supplementary Fig. 6a). Considering that Ca^2+^ was more effective than Mg^2+^ in *D. grandis* spheroplast enlargement^7^, we prepared a new broth, eMMB3CaKMg (62 mM CaCl_2_, 7.4 mM KCl, and 16.2 mM MgCl_2_), which was a modification of MMB3CaKMg (Supplementary Table 1). As a result, the enlargement effect was higher in eMMB3CaKMg than MMB3CaKMg (Supplementary Fig. 6). Although in MMB3CaKMg the spheroplasts began to collapse within 24 h of incubation, their enlargement continued after 24 h in eMMB3CaKMg (Supplementary Fig. 6). Thus, we used enlarged cells incubated in MMB3CaKMg and eMMB3CaKMg for microinjection. The enlarged spheroplasts in eMMB3CaKMg had the largest plasma membrane size and formed the highest number of vacuoles, which had various sizes (Supplementary Fig. 6b). These results showed that metal salt composition affects plasma membrane expansion and generation of vacuoles.

### Morphology comparison of *E. faecalis* and *L. amnigena* enlarged cells

**Figure 2:**
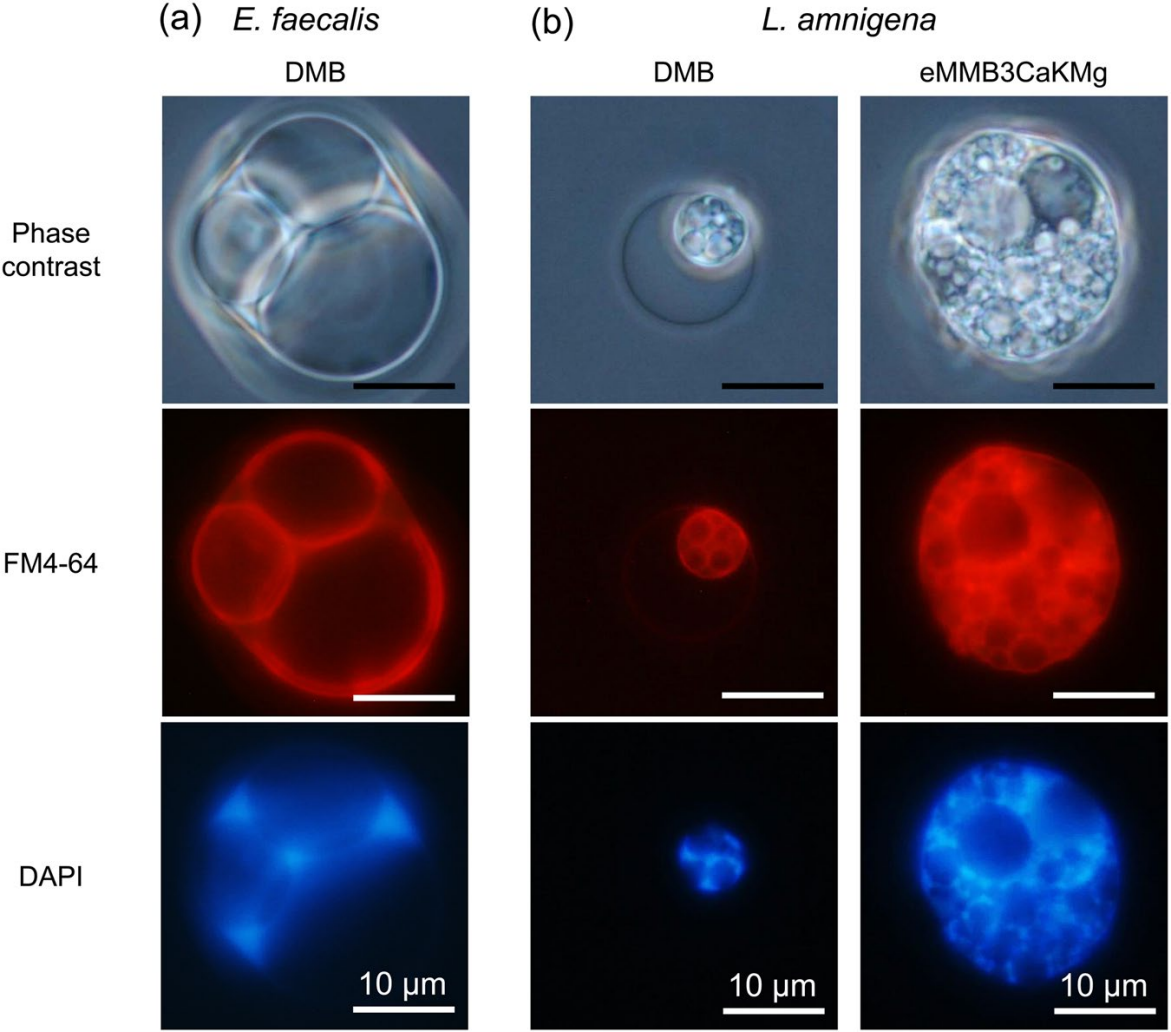
Microscopy images of *E. faecalis* and *L. amnigena* enlarged cells. (**a**) *E. faecalis* enlarged protoplasts after 144 h of incubation in DMB containing penicillin G. (**b**) *L. amnigena* enlarged spheroplasts after 20 h of incubation in DMB and eMMB3CaKMg containing penicillin G. The membrane and DNA were labelled with FM4-64 and DAPI, respectively. Phase contrast and fluorescent microscopy images were captured using an Olympus BX51 microscope. Scale bar = 10 µm.

**Figure 3:**
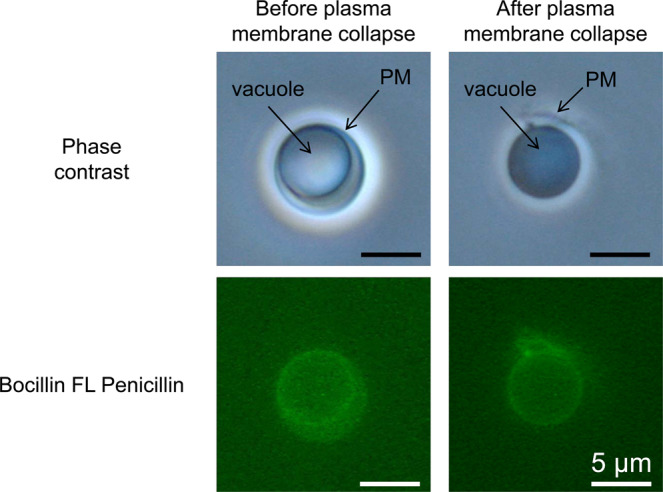
Bocillin FL penicillin staining. *E. faecalis* enlarged protoplasts after 72 h of incubation in DMB containing penicillin G. The penicillin binding protein was labelled with Bocillin FL penicillin. Phase contrast and fluorescent microscopy images were captured using an Olympus BX51 microscope. PM, plasma membrane. Scale bar = 5 µm.

An optical microscope observation revealed that the vacuoles observed in the cytoplasm of the *E. faecalis* and *L. amnigena* cells had diameters larger than approximately 10 μm (Supplementary Fig. 7). To elucidate the characteristics of *E. faecalis* and *L. amnigena* enlarged cells, DNA and membrane-staining experiments were performed (Fig. 2). Spherical vacuolar membranes in the cytoplasm were stained as well as the plasma membranes by FM4-64 in both *E. faecalis* and *L. amnigena* enlarged cells (Fig. 2). In addition, spherical vacuole membranes were also stained by Bocillin FL penicillin in *E. faecalis*, indicating vacuolar membranes contain a penicillin binding protein^13^ as well as the plasma membrane (Fig. 3). We confirmed that no vacuoles from *E. faecalis* and *L. amnigena* contained any DNA (Fig. 2). Nucleoids were located in the cytoplasm outside of the vacuoles (Fig. 2). These results are consistent with the characteristics of the vacuoles in the enlarged *E. coli* spheroplasts^2^ and *B. subtilis* protoplasts^3^. Thus, the vacuolar membranes of *E. faecalis* and *L. amnigena* probably have the same or similar components to the plasma membranes.

**Figure 4:**
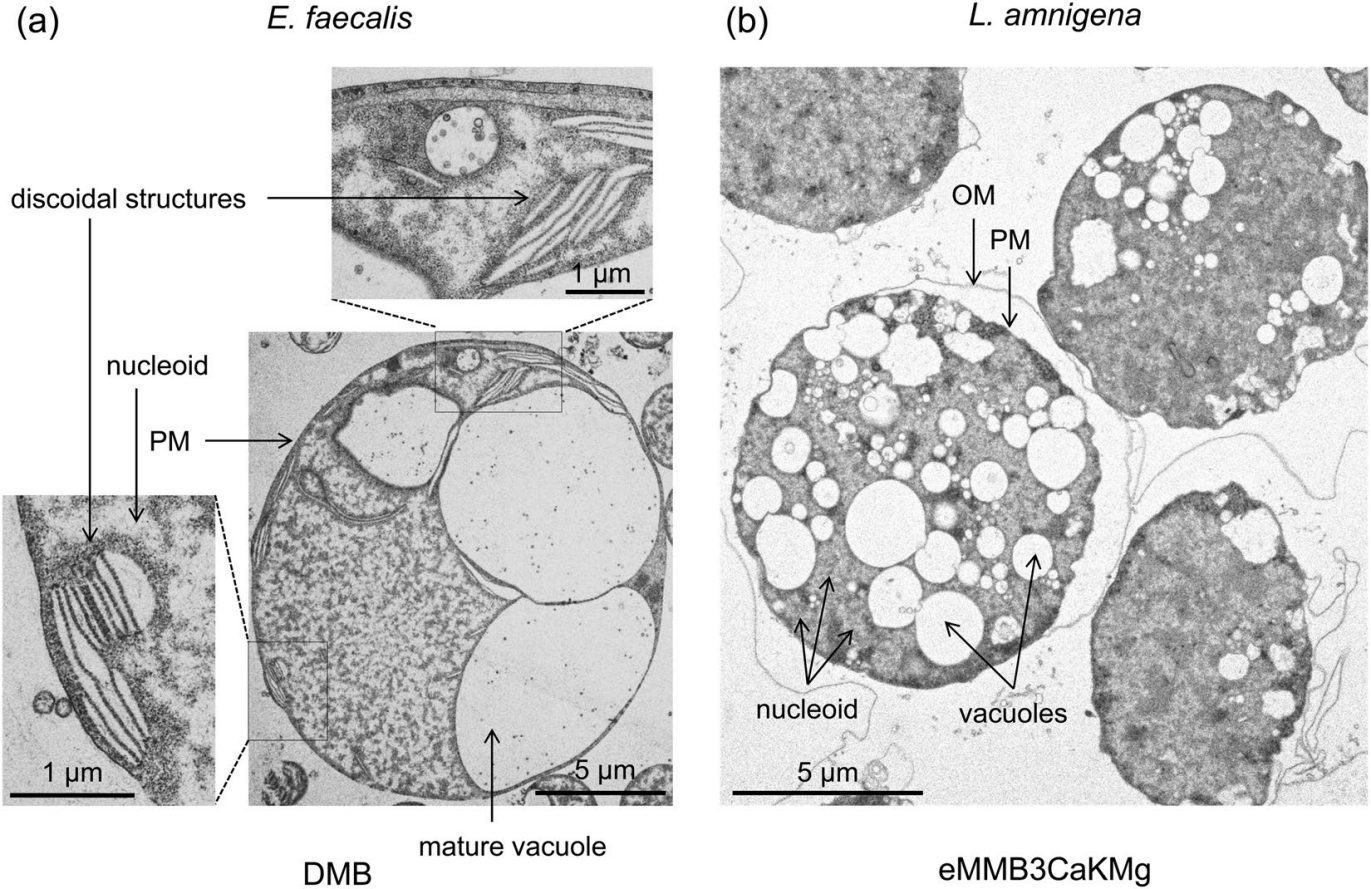
Transmission electron microscopy images of *E. faecalis* and *L. amnigena* enlarged cells. (**a**) *E. faecalis* enlarged protoplast after 65 h of incubation in DMB containing penicillin G. (**b**) *L. amnigena* enlarged spheroplast after 24 h of incubation in eMMB3CaKMg containing penicillin G. OM, outer membrane; PM, plasma membrane. Scale bars = 1 µm and 5 µm for panels a and b, respectively.

**Figure 5:**
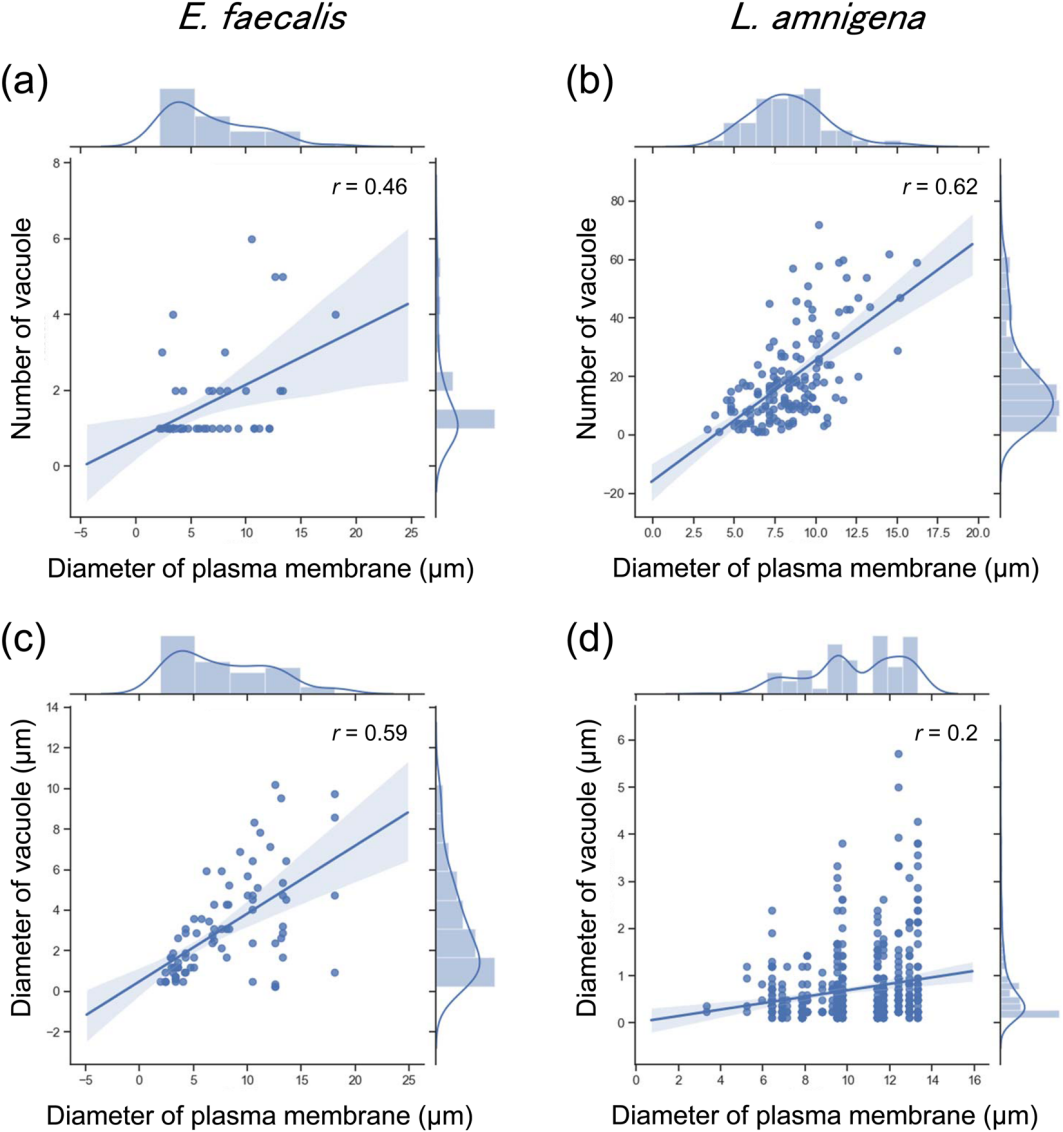
Relationships between cell size and vacuole synthesis in *E. faecalis* protoplasts and *L. amnigena* spheroplasts. The diameter of the plasma membrane and vacuole and the number of vacuoles were measured using transmission electron microscopy images of the *E. faecalis* protoplasts at 65 h of incubation and the *L. amnigena* spheroplasts at 24 h of incubation. (**a**) Relation between the diameter of the plasma membrane and the number of vacuoles in *E. faecalis* (*n* = 48). (**b**) Relation between the diameter of the plasma membrane and the number of vacuoles in *L. amnigena* (*n* = 154). (**c**) Relation between the diameter of the plasma membrane and the diameter of vacuoles in *E. faecalis* (cell; *n* = 48, vacuole; *n* = 82). (**d**) Relation between the diameter of the plasma membrane and the diameter of vacuoles in *L. amnigena* (cell; *n* = 19, vacuole; *n* = 506).

Based on the transmission electron microscopy images of the *E. faecalis* protoplasts at 65 h post-incubation and the *L. amnigena* spheroplasts at 24 h post-incubation, we measured the diameters of cells and vacuoles and counted the number of vacuoles in each cytoplasm (Fig. 4). These results were represented by scatter plot and histogram (Fig. 5). The diameter of cells and the number of vacuoles had a higher correlation in *L. amnigena* (*r* = 0.62, *n* = 154) than *E. faecalis* (*r* = 0.46, *n* = 48) (Fig. 5a,b). On the other hand, the diameter of cells and the diameter of vacuoles had a higher correlation in *E. faecalis* (*r* = 0.59, cell; *n* = 48, vacuole; *n* = 82) than *L. amnigena* (*r* = 0.2, cell; *n* = 19, vacuole; *n* = 506) (Fig. 5c,d). These results showed that in the enlargement process of the spheroplasts and protoplasts, the *E. faecalis* vacuoles increased the size and the *L. amnigena* vacuoles increased in number.

The transmission electron microscopy images showed many discoidal structures in enlarged *E. faecalis* protoplasts (Fig. 4a). The discoidal structures could not be observed by phase contrast microscopy (Fig. 2a and Supplementary Fig. 7a). Such structures have not been observed in enlarged *L. amnigena* cells (Fig. 4b) or in any bacterial cells^2,3,7^.

### Permeability of vacuolar membrane

In order to evaluate whether the enlarged protoplasts and spheroplasts can be used for microinjection, a blue fluorescent protein (BFP) solution was microinjected into the cytoplasm of the *E. faecalis* protoplasts and *L. amnigena* spheroplasts using an Eppendorf TransferMan 4r micromanipulator (Supplementary Fig. 8).

**Figure 6:**
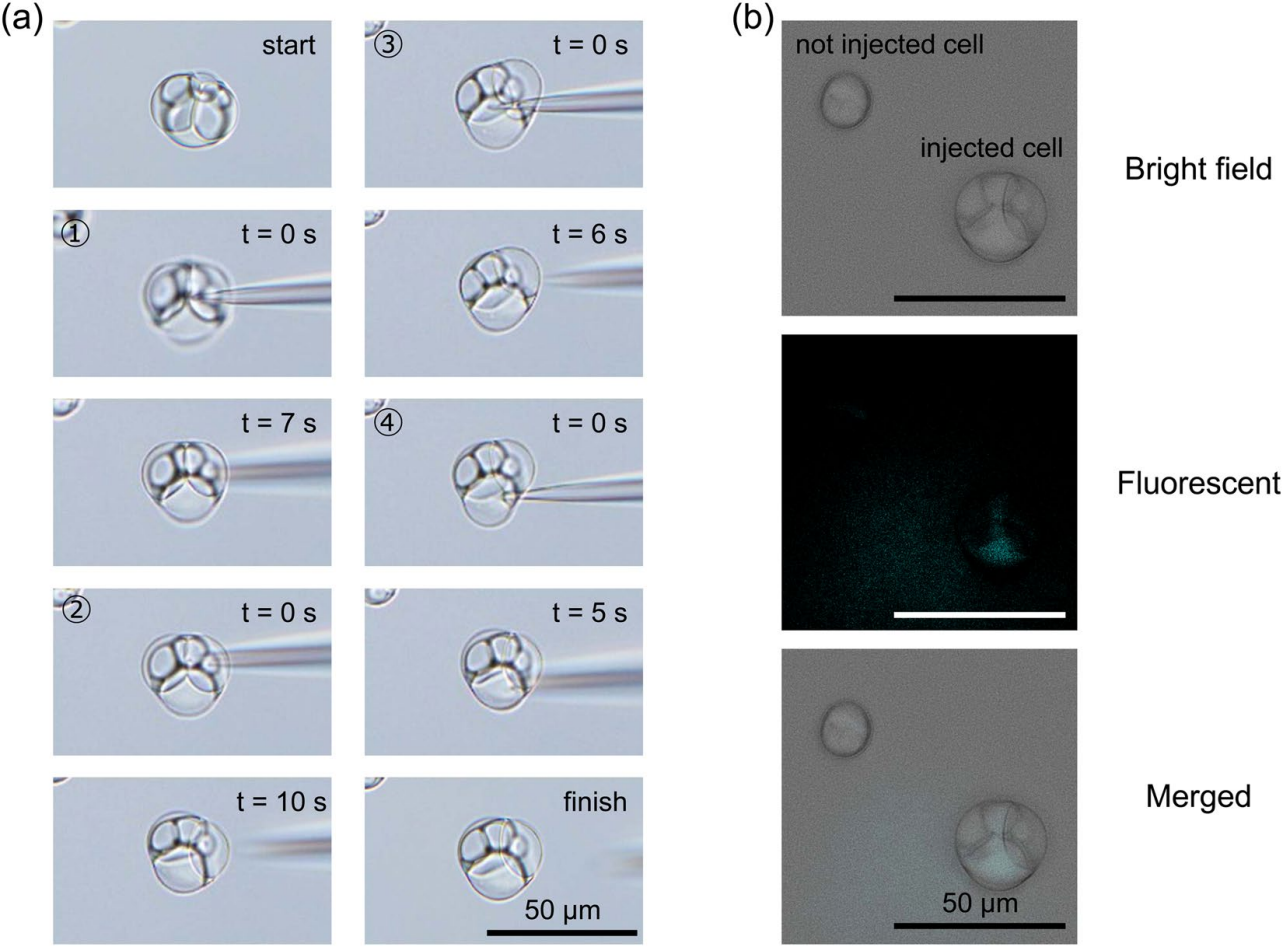
Microinjection of fluorescent protein solution into *E. faecalis* enlarged protoplast. The protoplasts were incubated for 96 h in DMB containing penicillin G. (**a)** Time-lapse images of microinjection. The start of injection was set to 0 s. The microinjection was performed a total of 4 times. (**b**) Fluorescent microscopy images of BFP. The figure includes microscopy images of microinjected protoplasts and non-microinjected protoplasts. Differential interference contrast microscopy images were captured using an Olympus IX73 microscope. Fluorescent microscopy images were captured using a Keyence BZ-X710 microscope. Scale bar = 50 µm.

First, we used the enlarged *E. faecalis* protoplasts incubated in DMB. The protoplasts were flexible and viscous; therefore, they adhered to the glass slide to perform the microinjection without use of a holding pipette (Fig. 6a and Supplementary Movie 1). The BFP solution was successfully microinjected into the cytoplasm of the remaining cell, as confirmed by fluorescence microscopy (Fig. 6b and Supplementary Fig. 9). In addition, fluorescence microscopy showed that BFP was contained in the cytoplasm but not in the vacuoles (Fig. 6b and Supplementary Fig. 9).

**Figure 7:**
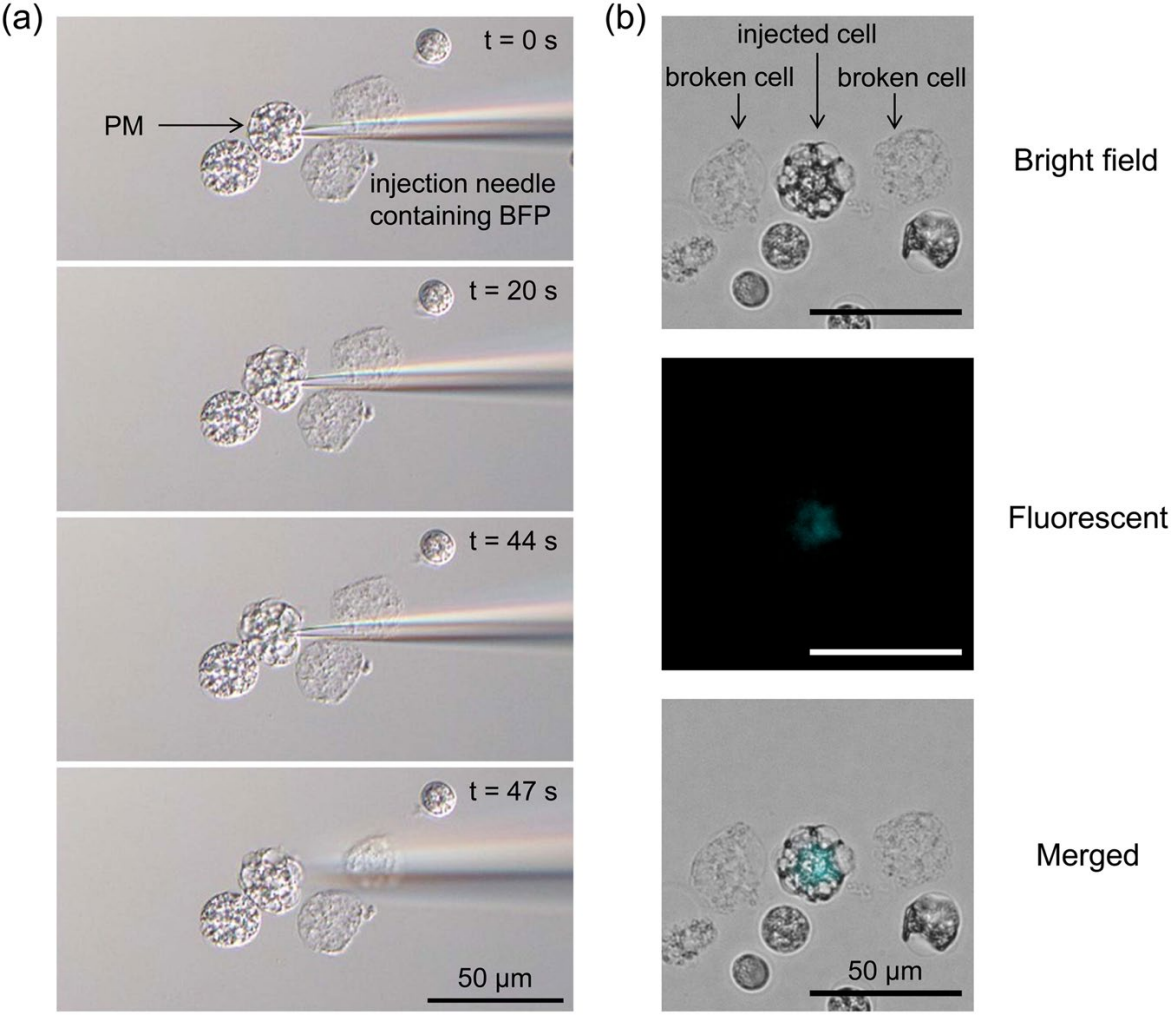
Microinjection of fluorescent protein solution into *L. amnigena* enlarged spheroplast. The spheroplasts were incubated for 28 h in eMMB3CaKMg containing penicillin G and surrounded only by the plasma membrane (protoplasts). (**a)** Time-lapse images of microinjection. The start of injection was set to 0 s. BFP solution was injected into the cytoplasm at 44 s. (**b**) Fluorescent microscopy images of BFP. The figure includes microscopy images of microinjected protoplasts and collapsed protoplasts. Differential interference contrast microscopy images were captured using an Olympus IX73 microscope. Fluorescent microscopy images were captured using a Keyence BZ-X710 microscope. PM, plasma membrane. Scale bar = 50 µm.

Next, we used enlarged *L. amnigena* spheroplasts incubated in eMMB3CaKMg. Because the spheroplasts were floating cells, a holding pipette was used (Supplementary Fig. 10). The outer membranes of the enlarged spheroplasts were removed using a microinjection-needle (Supplementary Fig. 10). The protoplasts were flexible and viscous; they adhered to the glass slide and microinjection could be performed without the use of a holding pipette (Fig. 7a and Supplementary Fig. 10 and Movie 2). The BFP solution was successfully microinjected into the cytoplasm, as confirmed by fluorescence microscopy (Fig. 7b). Fluorescence microscopy showed that BFP was contained in the cytoplasm but not in the vacuoles, and no fluorescence was observed in the debris of cells broken during the injection (Fig. 7b). These results showed that both *E. faecalis* and *L. amnigena* enlarged cells could be used for microinjection and the vacuolar membranes were devoid of the fluorescent protein.

### Flexibility of plasma membrane

**Figure 8:**
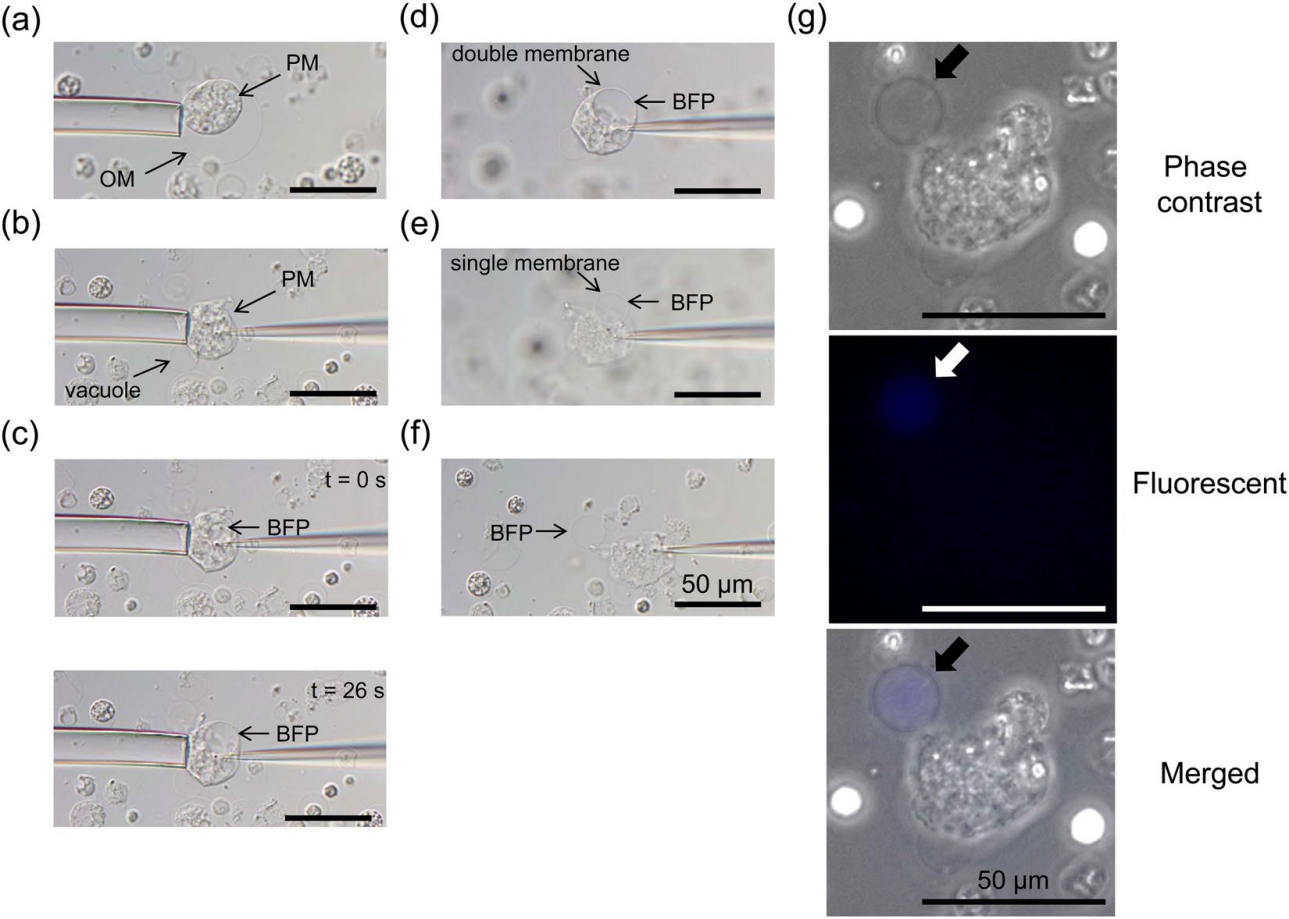
Generation of vacuole containing fluorescent protein using the micromanipulator. The spheroplasts were incubated for 20 h in MMB3CaKMg containing penicillin G. (**a**) Fixation of *L. amnigena* enlarged spheroplasts by a holding pipette. The spheroplasts displayed plasma and outer membrane. (**b**) Removal of the outer membrane by insertion of the microinjection needle followed by the appearance of the vacuole. (**c**) Time-lapse images of generation and enlargement of vacuole containing BFP. (**d**) Final stage in the generation of BFP-containing vacuole. (**e**) Collapse of protoplast followed by the appearance of the vacuole containing BFP. (**f**) Isolation of the vacuole containing BFP. (**g**) Fluorescent microscopy images of BFP. Differential interference contrast microscopy images were captured using an Olympus IX73 microscope. Fluorescent microscopy images were captured using a Keyence BZ-X710 microscope. OM, outer membrane; PM, plasma membrane. Scale bar = 50 µm.

Endocytosis is a system that takes up extracellular material by invaginating the plasma membrane. In our microinjection experiments, an artificial vacuole was generated by releasing BFP solution into the plasma membrane of enlarged *L. amnigena* spheroplasts (Fig. 8 and Supplementary Movie 3). We used enlarged *L. amnigena* spheroplasts incubated in MMB3CaKMg. After removing the outer membrane by inserting the microinjection-needle, we brought the microinjection-needle close to the protoplast while releasing the BFP solution (Fig. 8a,b). When the microinjection-needle contacted the protoplast, the plasma membrane expanded inside like a balloon and subsequently a vacuole was generated in the cell (Fig. 8c). The vacuole enlarged from 13.9 µm in diameter to 18.8 µm in diameter during a 26 second interval (Fig. 8c). Thus, BFP solution was being released at a rate of approximately 0.08 pl s^−1^ from the tip of the microinjection-needle. This vacuole existed after the microinjection needle was pulled out (Fig. 8d). Finally, the protoplast was collapsed (Fig. 8e). Surprisingly, after the protoplast collapse, the vacuole was maintained in the solution, indicating that this vacuole was not connected to the plasma membrane (Fig. 8f). The fluorescent microscope observation showed that the BFP existed in this vacuole (Fig. 8g). Thus, the plasma membrane of the enlarged *L. amnigena* spheroplast incubated in MMB3CaKMg has such significant flexibility and viscosity that the vacuole could be created using micromanipulator.

On the other hand, no artificial vacuoles were generated in the microinjection experiments using the enlarged *E. faecalis* protoplasts. This result showed that the high plasma membrane flexibility of *L. amnigena* allowed for artificial vacuole formation.

## Discussion

For microinjection into cells, the cell size should be more than 15 μm in diameter. All of the enlarged spheroplasts and protoplasts used in microinjection have vacuoles in the cytoplasm of bacterial cells. Our findings indicated that vacuole synthesis continued during cell enlargement. These results imply that vacuole generation and synthesis are essential for bacterial spheroplast and protoplast enlargement and that plasma membrane expansion is associated with vacuolar membrane synthesis in bacterial protoplast enlargement. On the other hands, vacuoles are always observed in the enlarged bacterial spheroplasts or protoplasts (cell size > approximately 10 μm in diameter), but their role is uncertain. Most bacteria produce ATP using ATP synthase on the plasma membrane. Thus, the volume of cytoplasm may be limited due to the plasma membrane size. Our hypothesis is that vacuole enlargement may inhibit the increase of the cytoplasm volume.

Results of the nucleoid, phospholipid and the penicillin binding protein localization using DAPI, FM4-64, and Bocillin FL penicillin staining, respectively confirmed that the fundamental characteristics of the *E. faecalis* and *L. amnigena* vacuoles were consistent with those of *E. coli* and *B. subtilis* enlarged cells. On the other hand, *Listeria* L-forms form intracellular vesicles as reproduction elements by local phospholipid accumulation or membrane invagination^14,15^. The vacuoles of *E. faecalis* and *L. amnigena* are not intracellular vesicles in the same way that reproduction elements are because they lack DNA and phospholipid accumulation.

In the enlarged protoplasts of *E. faecalis*, spherical and discoidal structures were observed within 65 h of incubation (Fig. 4a). Time-lapse observation using bright field microscopy showed that a newly formed small vacuole has grows more quickly than the previously formed vacuole(s), strongly suggesting that the expansion speed of the smaller vacuole is faster than that of larger vacuoles in the enlargement process of *E. faecalis* protoplasts until those vacuoles reach the same size (Fig. 1). If the discoidal structures are precursors to spherical vacuoles, the vacuole generation is continuously occurring even after 65 h of incubation. We observed multiple small vacuoles when a microinjection needle was inserted into the *E. faecalis* protoplast (Supplementary Fig. 11). The discoidal structure may absorb the water of the injected solution and then transform into a spherical structure, we think.

We have shown that the enlarged cells suitable for microinjection could be produced by manipulating the metal salts amounts in the medium (Supplementary Figs. 3 and 5). In microinjection process, the *L. amnigena* enlarged cells incubated in DMB could not be used but the cells incubated in eMMB3CaKMg could be used, indicating that the metal ions changed the membrane characteristics. On the other hand, *E. faecalis* enlarged cells incubated in DMB could be used for microinjection, indicating that metal salts composition of DMB is suitable for forming micro-injectable membrane in *E. faecalis*. *E. faecalis* and *L. amnigena* showed that the fluorescent protein did not pass through the vacuolar membrane from the cytoplasm, which indicates that the vacuolar membrane has a substance-selecting function. In addition, the vacuolar membranes were stained by Bocillin FL penicillin, indicating that a penicillin binding protein exists in the vacuolar membrane as well as the plasma membrane. This suggests that the vacuolar membrane has similar components to the plasma membrane in *E. faecalis* and *L. amnigena*. However, the characteristics of *E. faecalis* and *L. amnigena* plasma membranes differed. In *L. amnigena*, when the microinjection needle was brought close to the plasma membrane while releasing the solution, the membrane invaded to the cytoplasm and endocytosis occurred (Fig. 8). By contrast, in *E. faecalis*, membrane invagination and endocytosis were never observed in the microinjection experiment. These results indicated that the membrane flexibility of *E. faecalis* is higher than that of *L. amnigena*. From the electron micrograph, the surface layer of *E. faecalis* is sharp, while that of *L. amnigena* is flaccid (Fig. 4). This supports our postulation that the *L. amnigena* plasma membrane is more flexible than the *E. faecalis* plasma membrane.

Medium components affect membrane properties in the bacterial cell enlargement process. In *D. grandis*, the metal salts and osmotic stabilizers affected the lipid composition of the membrane and the frequency of outer membrane fusion in the enlargement process^7,16,17^. Based on the previous studies and this study, the composition of metal salts in the incubation media affects the membrane synthesis during the bacterial spheroplast or protoplast enlargement. However, the metal salts that play an important role in cell enlargement differ among the bacterial species. For example, although enlargement of *Deinococcus* spheroplasts requires calcium ion or magnesium ion^7^, that of *Lelliottia* does not. Thus, the type of the metal salt varies depending on the bacterial species. Interestingly, the phase contrast microscope observation showed that *D. grandis* enlarged spheroplasts, which inhibited the plasma membrane expansion, do not generate vacuoles in the cytoplasm^7,16,17^. Thus, outer membrane expansion is not associated with vacuole generation. In contrast to this, our findings showed that composition and concentration of metal salts affect not only outer membrane but also plasma membrane expansion during the enlargement of *L. amnigena* spheroplasts. In addition, *L. amnigena* spheroplasts with larger plasma membranes had more vacuoles in the cytoplasm (Fig. 5b). This indicates that the *L. amnigena* plasma membrane synthesis is accompanied by vacuolar membrane synthesis in the process of the spheroplast enlargement. However, in the process of *E. faecalis* protoplast enlargement, each vacuole also became larger (Fig. 5c). This was confirmed by time-lapse observation using the bright field microscope (Fig. 1). Thus, the plasma membrane synthesis is also accompanied by vacuolar membrane synthesis in the *E. faecalis* protoplast enlargement.

The *E. faecalis* protoplasts and *L. amnigena* spheroplasts exhibit different types of vacuole generation during the cell enlargement. However, the biosynthesis of vacuolar and plasma membranes occurs at the same time during enlargement. At the early stage of cell enlargement, no vacuoles are generated^8^. In addition, spheroplasts of the purple bacteria *Erythrobacter* and *Rhodospirillum* could not generate vacuole were enlarged to a maximum of 7 μm in diameter^5,6^. In the *E. faecalis* protoplast enlargement, inhibition of the plasma membrane expansion leads to inhibition of vacuole generation^8^.

Moreover, vacuole of *E. coli* is morphologically similar to that of *L. amnigena*, and that of *B. subtilis* is similar to that of *E. faecalis*. The difference between gram-negative and gram-positive may affect vacuole generation. On the other hands, the number of vacuoles of *E. coli* is smaller than that of *L. amnigena*, and the vacuole of *B. subtilis* has less force to push the cell membrane than that of *E. faecalis*. As mentioned above, the enlarged gram-negative *D. grandis* has larger outer membrane than plasma membrane and does not generate vacuoles, which completely differs from *E. coli* and *L. amnigena*^7^. Thus, the vacuole type has a morphological variation among different species of bacteria. The vacuole type may depend on the plasma membrane character. The vacuoles may function in the enlarged cells, since the vacuolar membrane has the plasma membrane proteins^2,3^. In addition, in nature, the largest bacterium, *Thiomargarita namibiensis* also generates an unusually large vacuole and accumulates nitrate in the vacuole^18,19^. The bacteria contained sulfur in the cytoplasm^18,19^. The vacuole function is the separation of nitrate and sulfide^18^. Our incubation medium for enlargement do not contain sulfur, therefore, the physiological function differs between the enlarged protoplasts and *T. namibiensis* large cells. In general, vacuoles are not observed in normal bacterial cells, suggesting that vacuole is not needed for bacterial normal growth. On the other hand, vacuole may be essential for maintaining giant bacterial cells.

## Methods

### Preparation and cultivation of spheroplast or protoplast

A single colony of *L. amnigena* NBRC105700 was streaked on an LB agar plate (10 g l^−1^ tryptone, 5.0 g l^−1^ yeast extract, 5 g l^−1^ sodium chloride, and 15 g l^−1^ Bacto agar obtained from BD, Franklin Lakes, NJ) and cultivated for one to two days at 30 °C. A single colony was then inoculated in 5 ml of fresh LB broth overnight at 30 °C. An aliquot (500 μl) of the overnight culture was resuspended in 10 ml of LB broth, and subsequently cultivated at 30 °C with shaking at 150 r min^−1^ until the culture reached an OD_600_ of 0.7. The cells were centrifuged at 11,000 × *g* for 1 min and then resuspended in Difco Marine Broth 2216 (DMB) (5 g l^−1^ peptone, 1 g l^−1^ yeast extract, 0.1 g l^−1^ ferric citrate, 19.45 g l^−1^ NaCl, 5.9 g l^−1^ MgCl_2_, 3.24 g l^−1^ MgSO_4_, 1.8 g l^−1^ CaCl_2_, 0.55 g l^−1^ KCl, 0.16 g l^−1^ NaHCO_3_, 0.08 g l^−1^ KBr, 34 mg l^−1^ SrCl_2_, 22 mg l^−1^ H_3_BO_3_, 8 mg l^−1^ Na_2_HPO_4_, 4 mg l^−1^ Na_2_SiO_3_, 2.4 mg l^−1^ NaF, and 1.6 mg l^−1^ NH_4_NO_3_ obtained from BD, Franklin Lakes, NJ) containing 300 μg ml^−1^ penicillin G (Wako, Osaka). Other media used were MMB0 (5 g l^−1^ peptone [BD, Franklin Lakes, NJ], 1 g l^−1^ yeast extract [BD, Franklin Lakes, NJ], 0.1 g l^−1^ ferric citrate [Sigma-Aldrich, St. Louis, MO]), MMB (MMB0 with 19.45 g l^−1^ NaCl [Nacalai, Kyoto], 5.9 g l^−1^ MgCl_2_ [Wako, Osaka], 1.8 g l^−1^ CaCl_2_ [Wako, Osaka], and 0.55 g l^−1^ KCl [Nacalai, Kyoto]), or MMB0 with different concentrations of metal salts, containing 300 μg ml^−1^ penicillin G^7^. The resulting suspension (4 μl) was diluted with 2 ml of the appropriate medium (DMB or MMB) and incubated at 24 °C.

A single colony of *E. faecalis* NBRC100480 was streaked on an MRS broth agar plate (10 g l^−1^ proteose peptone No. 3, 10 g l^−1^ beef extract, 5.0 g l^−1^ yeast extract, 20 g l^−1^ dextrose, 1.0 g l^−1^ polysorbate 80, 2.0 g l^−1^ ammonium citrate, 5.0 g l^−1^ sodium acetate, 0.1 g l^−1^ magnesium sulphate, 0.05 g l^−1^ manganese sulphate, 2.0 g l^−1^ dipotassium phosphate, and 15 g l^−1^ Bacto agar obtained from BD, Franklin Lakes, NJ) and cultivated for one to two days at 37 °C. A single colony was then inoculated in 5 ml of fresh MRS broth overnight at 37 °C. An aliquot (500 μl) of the overnight culture was resuspended in 10 ml of MRS broth, and subsequently cultivated at 37 °C with no shaking until the culture reached an OD_600_ of 0.7. The cells (1 ml) were centrifuged at 11,000 × *g* for 1 min and then resuspended in a buffer (1 ml) consisting of 0.1 M Tris-HCl (pH 7.6) and 0.3 M sucrose containing 5 mg ml^−1^ labiase (Cosmo Bio, Tokyo)^20^ or 5 mg ml^−1^ egg white lysozyme (Wako, Osaka) (Fig. S1d). The mixture was incubated at 37 °C with no shaking for 3 h. Protoplasts were centrifuged at 7000 r.p.m. for 5 min and resuspended in DMB containing 300 µg ml^−1^ penicillin G. Other media used were MMB0, MMB, or MMB0 with different concentrations of metal salts, containing 300 μg ml^−1^ penicillin G. Penicillin was added to inhibit cell wall regeneration in protoplasts. The resulting suspension (10 μl) was diluted with 2 ml of the appropriate medium (DMB or MMB) and incubated at 24 °C.

### Membrane and DNA staining

To acquire the fluorescence images of the membrane, the *L. amnigena* spheroplasts or *E. faecalis* protoplasts were mixed with FM4-64 (Invitrogen, USA) and DAPI (Dojindo, Kumamoto) at final concentrations of 5.0 and 0.5 µM, respectively, and then incubated for 10 min at 24 °C. To acquire the fluorescence images of the penicillin binding protein, the *E. faecalis* protoplasts were mixed with Bocillin FL penicillin (Invitrogen, USA)^13^ at final concentrations of 10 µg ml^−1^, and then incubated for 10 min at 24 °C. Phase contrast and fluorescent microscope images were captured using an Olympus BX51 (Olympus, Japan) microscope.

### Transmission electron microscopy

Transmission electron microscopy was carried out according to the procedure described previously^17^.

### Microinjection workstation

The microinjection workstation consisted of an Olympus IX73 (Olympus, Japan) differential interference microscope with a UPlanFL N objective (4×), a UPlanFL N objective (10×) and an LUCPlan FL N objective (40×). The microscope was equipped with a TransferMan 4r (Eppendorf, Germany) micromanipulator set with the manual microinjectors CellTram Air and CellTram vario (Eppendorf, Germany).

### Loading the blue fluorescent protein injection solution into the microinjection needle

The blue fluorescent protein (BFP, Wako, Osaka) injection solution was centrifuged at 11,000 × *g* for 15 min at 4 °C and a 1 µl aliquot was loaded into the tip of a Femtotip II microinjection needle (pore size: 0.5 µm, Eppendorf, Germany). Next, the needle was tightly mounted in the capillary holder of a microinjector CellTram vario, and then fixed onto the micromanipulator.

### Microinjection of *L. amnigena* and *E. faecalis* enlarged cells

The *L. amnigena* enlarged spheroplast culture or *E. faecalis* enlarged protoplast culture were mounted on a glass slide, and placed on the microscope stage. A Piezo Drill Tip ES (pore size: 15 µm, Eppendorf, Germany) was tightly mounted in the capillary holder of a microinjector Cell Tram Air, and then fixed onto the micromanipulator. The enlarged cells were fixed by the Piezo Drill Tip ES. The BFP solution was released into the enlarged cells using a CellTram vario microinjector. The needle was inserted into the enlarged cells while releasing the BFP solution. The glass slide was examined under a BZ-X710 (Keyence, Japan) fluorescence microscope, and successful injection was confirmed by fluorescence detection.

## Supplementary information

Supplementary Information.

Supplementary Information2.

Supplementary Information3.

Supplementary Information4.

Supplementary Information5.
