# Supplementary material for: Species-dependent protoplast enlargement involves different types of vacuole generation in bacteria
Source: Sci Rep. 2020 Jun 1;10:8832. doi: 10.1038/s41598-020-65759-7 (PMC7264237; doi:10.1038/s41598-020-65759-7)
Supplement: Supplementary file 1 — Supplementary Information. [file 41598_2020_65759_MOESM1_ESM.pdf]

Species-dependent protoplast enlargement involves different types of vacuole generation in bacteria

Sawako Takahashi*, Marin Mizuma, Satoshi Kami, Hiromi Nishida*

Biotechnology Research Center and Department of Biotechnology, Toyama Prefectural University, 5180 Kurokawa, Imizu, Toyama 939-0398, Japan

*Correspondence should be addressed to S. T. (t876002@st.pu-toyama.ac.jp) or H. N. (hnishida@pu-toyama.ac.jp).

Supplementary Data Legends

Supplementary Table 1. Modified Marine Broth used in this study.

Supplementary Figure 1. Characterization of *L. amnigena* and *E. faecalis* cells. NHS-Alexa Fluor 488 (Invitrogen, USA) was used to stain outer membrane proteins of *L. amnigena* before lysozyme treatment[1]. (a) Walled, rod-shaped native forms of *L. amnigena*. (b) *L. amnigena* enlarged spheroplast after 48 h of incubation in DMB containing penicillin G. (c) Walled, spherical-shaped native forms of *E. faecalis*. (d) Protoplasts of *E. faecalis* immediately after labiase or lysozyme treatment. Phase contrast and fluorescent microscopy images were captured using an Olympus BX51 microscope. Scale bar = 5 µm.

Supplementary Figure 2. Characterization of enlarged spheroplasts of *L. amnigena*. (a) Differential interference contrast microscopy image of the enlarged spheroplasts of *L. amnigena* in DMB containing penicillin G after 72 h of incubation. The cell was enlarged using a previously described method[2]. (b) Differential interference contrast microscopy image of enlarged spheroplasts of *L. amnigena* in MMB3CaKMg at 20 h of incubation. Differential interference contrast microscopy images were captured using an Olympus IX73 microscope. Scale bar = 50 µm.

Supplementary Figure 3. Phase contrast microscopy images of *E. faecalis* protoplasts in modified marine broths (MMB). The protoplasts were incubated for 120 h in each medium containing penicillin G. Phase contrast microscopy images were captured using an Olympus CK X41 (Olympus, Japan) microscope. Scale bar = 50 µm.

Supplementary Figure 4. Phase contrast microscopy images of *E. faecalis* protoplasts in MMB1Ca, MMB1Mg, MMB1K and MMB1Na containing different concentrations. The protoplasts were incubated for 120 h in each medium containing penicillin G. Phase contrast microscopy images were captured using an Olympus CK X41 microscope. Scale bar = 50 µm.

Supplementary Figure 5. Phase contrast microscopy images of *L. amnigena* spheroplasts in modified marine broths (MMB). The spheroplasts were incubated for 20 h in each medium containing penicillin G. Phase contrast microscopy images were captured using an Olympus CK X41 microscope. Scale bar = 50 µm.

Supplementary Figure 6. Phase contrast microscopy images of *L. amnigena* spheroplasts in MMB3CaKMg and eMMB3CaKMg. The spheroplasts were incubated for 24 h and 48 h in each medium containing penicillin G. Phase contrast microscopy images were captured using an Olympus CK X41 microscope. Scale bar = 50 µm.

Supplementary Figure 7. An optical microscopy images of vacuoles in *E. faecalis* and *L. amnigena*. (a) Phase contrast microscopy images of enlargement of *E. faecalis* protoplasts in DMB containing penicillin G. The protoplasts were incubated for 48 h. (b) Differential interference contrast microscopy images of enlargement of *L. amnigena* spheroplasts in eMMB3CaKMg containing penicillin G. The spheroplasts were incubated for 16 h. Phase contrast microscopy images were captured using an Olympus CK X41 microscope. Differential interference contrast microscopy images were captured using an Olympus IX73 microscope. Scale bar = 10 µm.

Supplementary Figure 8. Micromanipulation system used in this study. (a) Photographic image of a TransferMan 4r (Eppendorf, Germany) micromanipulator set with the manual microinjectors CellTram Air and CellTram vario (Eppendorf, Germany). (b) Image of the microinjection workspace. (c) Schematic drawing illustrating the microinjection into an enlarged cell. The holding pipette (Piezo Drill Tip ES) and microinjection needle (Femtotip II) have pore sizes of 15 µm and 0.5 µm, respectively.

Supplementary Figure 9. *E. faecalis* enlarged protoplast microinjected BFP solution. The protoplasts were incubated for 96 h in DMB containing penicillin G. Bright field and fluorescent microscopy images were captured using a Keyence BZ-X710 microscope. Scale bar = 50 µm.

Supplementary Figure 10. Micromanipulation of *L. amnigena* enlarged spheroplasts. The spheroplasts were incubated for 24 h in eMMB3CaKMg containing penicillin G. The figure represents the time-lapse images of microinjection, removal of outer membrane by microinjection-needle, protoplast adhesion to the glass slide, and microinjection without the use of a holding pipette. The start of injection was set to 0 s. Differential interference contrast microscopy images were captured using an Olympus IX73 microscope. OM, outer membrane; PM, plasma membrane. Scale bar = 50 µm.

Supplementary Figure 11. Vacuole formation by microinjection in *E. faecalis* enlarged protoplasts. The protoplasts were incubated for 96 h in DMB containing penicillin G. The start of injection was set to 0 s. Differential interference contrast microscopy images were captured using an Olympus IX73 microscope. Scale bar = 50 µm.

Supplementary Video 1. Video showing the microinjection of BFP solution into the *E. faecalis* enlarged protoplasts. The protoplasts were incubated for 96 h in DMB containing penicillin G. Differential interference contrast microscopy images were captured using an Olympus IX73 microscope. Scale bar = 50 µm.

Supplementary Video 2. Video showing the microinjection of BFP solution into the *L. amnigena* enlarged protoplasts. The spheroplasts were incubated for 28 h in eMMB3CaKMg containing penicillin G and were covered only with plasma membrane (protoplast). Differential interference contrast microscopy images were captured using an Olympus IX73 microscope. Scale bar = 50 µm.

Supplementary Video 3. Video showing the generation of vacuole like structure including fluorescent protein using the micromanipulator. The spheroplasts were incubated for 20 h in MMB3CaKMg containing penicillin G. Differential interference contrast microscopy images were captured using an Olympus IX73 microscope. Scale bar = 50 µm.

Name	Componets	Strains	Source or reference
MMB0	5 g/l peptone, 1 g/l yeast extract, 0.1 g/l ferric citrate	L. a／E. f	14
MMB1Ca	MMB0, 16.2 mM $CaCl_2$	L. a／E. f	
MMB1K	MMB0, 7.4 mM KCl	L. a／E. f	
MMB1Mg	MMB0, 62 mM $MgCl_2$	L. a／E. f	
MMB1Na	MMB0, 333 mM NaCl	L. a／E. f	
MMB2CaK	MMB0, 16.2 mM $CaCl_2$, 7.4 mM KCl	L. a／E. f	
MMB2CaMg	MMB0, 16.2 mM $CaCl_2$, 62 mM $MgCl_2$	L. a／E. f	
MMB2CaNa	MMB0, 16.2 mM $CaCl_2$, 333 mM NaCl	L. a／E. f	
MMB2KMg	MMB0, 7.4 mM KCl, 62 mM $MgCl_2$	L. a／E. f	
MMB2KNa	MMB0, 7.4 mM KCl, 333 mM NaCl	L. a／E. f	
MMB2MgNa	MMB0, 62 mM $MgCl_2$, 333 mM NaCl	L. a／E. f	
MMB3CaKMg	MMB0, 16.2 mM $CaCl_2$, 7.4 mM KCl, 62 mM $MgCl_2$	L. a／E. f	
MMB3CaKNa	MMB0, 16.2 mM $CaCl_2$, 7.4 mM KCl, 333 mM NaCl	L. a／E. f	
MMB3CaMgNa	MMB0, 16.2 mM $CaCl_2$, 62 mM $MgCl_2$, 333 mM NaCl	L. a／E. f	
MMB3KMgNa	MMB0, 7.4 mM KCl, 62 mM $MgCl_2$, 333 mM NaCl	L. a／E. f	
MMB	MMB0, 16.2 mM $CaCl_2$, 7.4 mM KCl, 62 mM $MgCl_2$, 333 mM NaCl	L. a／E. f	
eMMB3CaKMg	MMB0, 62 mM $CaCl_2$, 7.4 mM KCl, 16.2 mM $MgCl_2$	L. a	This study

Table. S1

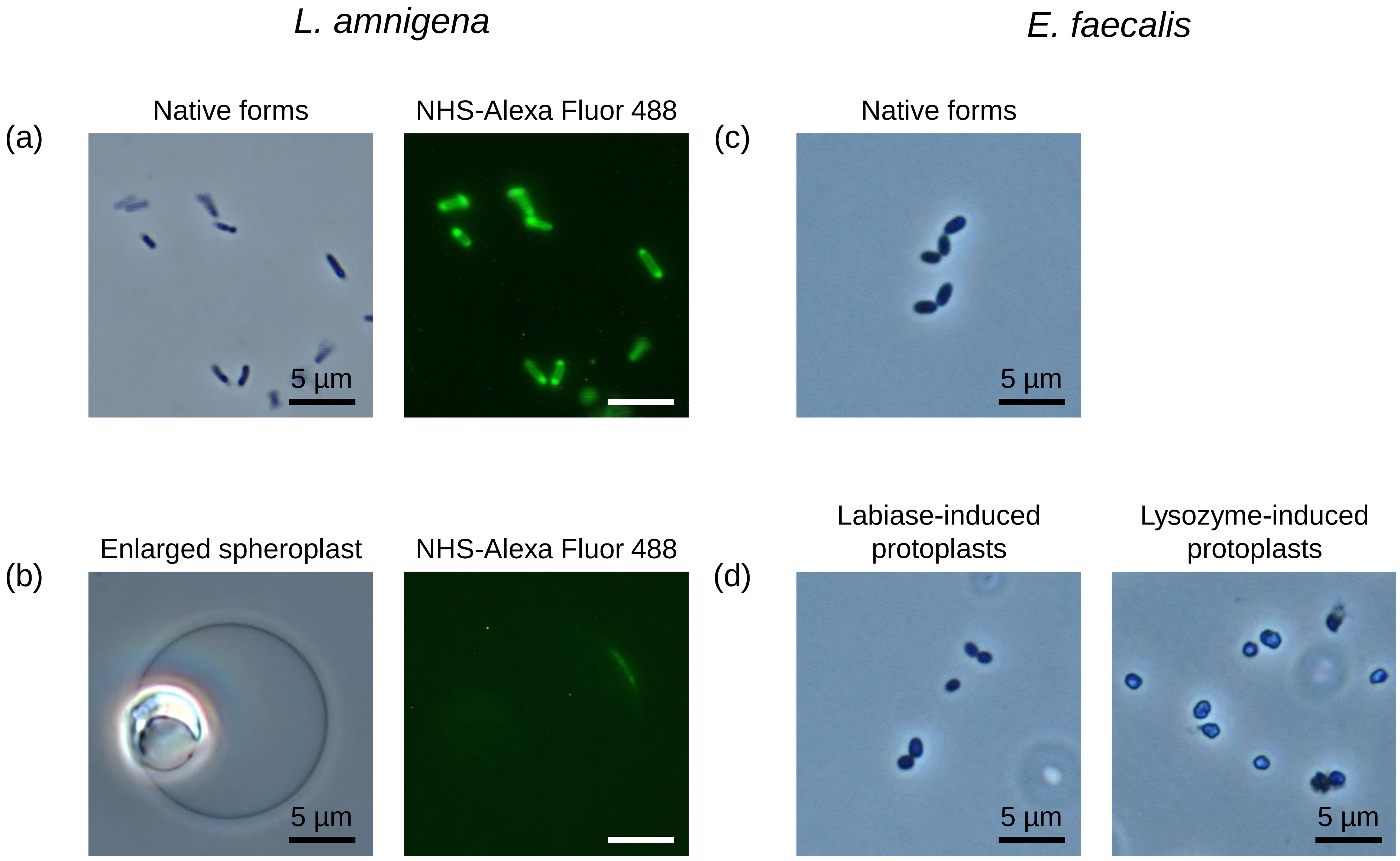
L. amnigena
E. faecalis
(a)
Native forms
NHS-Alexa Fluor 488
5 µm
(b)
Enlarged spheroplast
NHS-Alexa Fluor 488
5 µm
(c)
Native forms
5 µm
(d)
Labiase-induced protoplasts
Lysozyme-induced protoplasts
5 µm
5 µm

Fig. S1

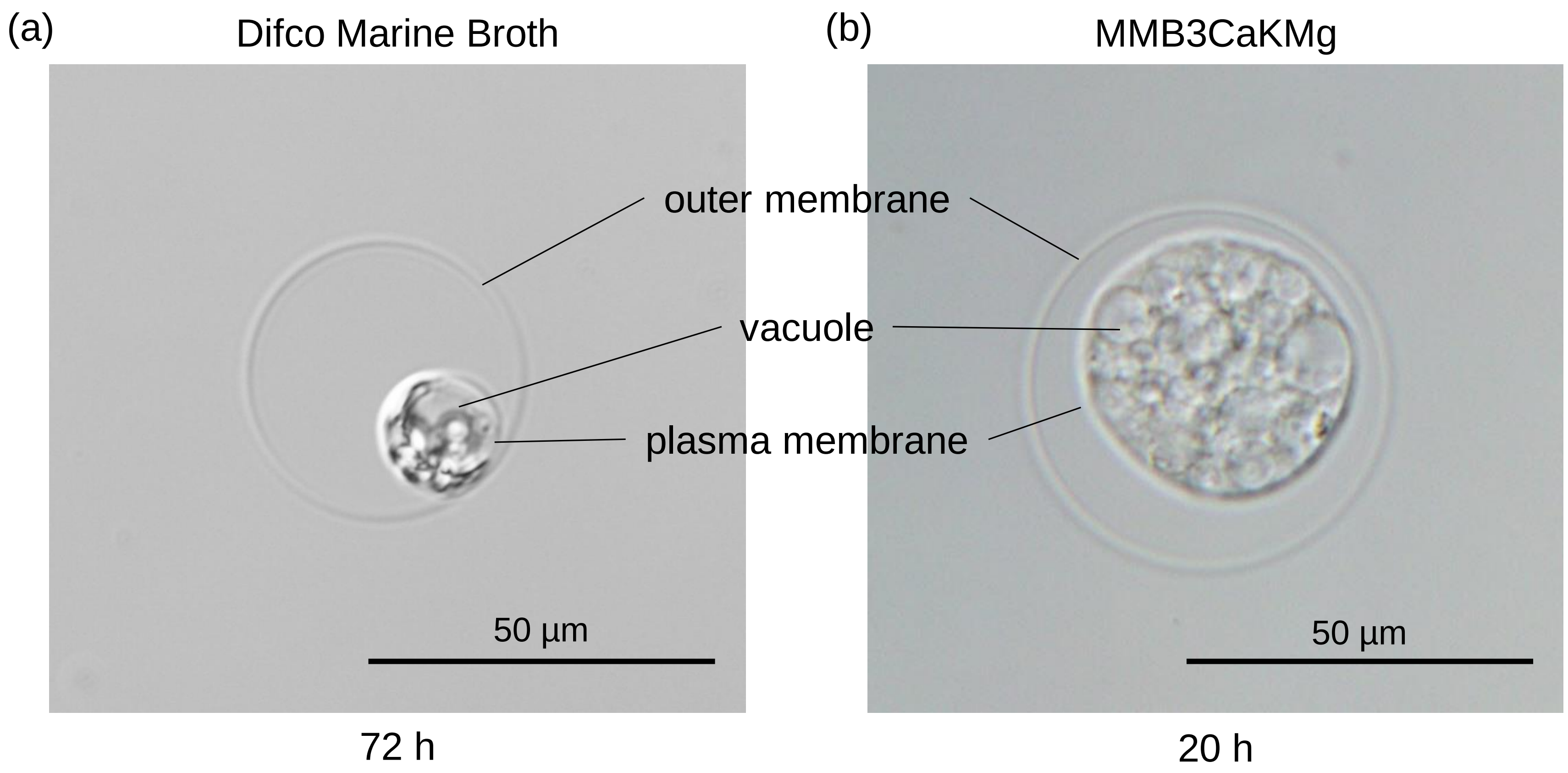
(a)
Difco Marine Broth
(b)
MMB3CaKMg
outer membrane
vacuole
plasma membrane
50 µm
50 µm
72 h
20 h

Fig. S2

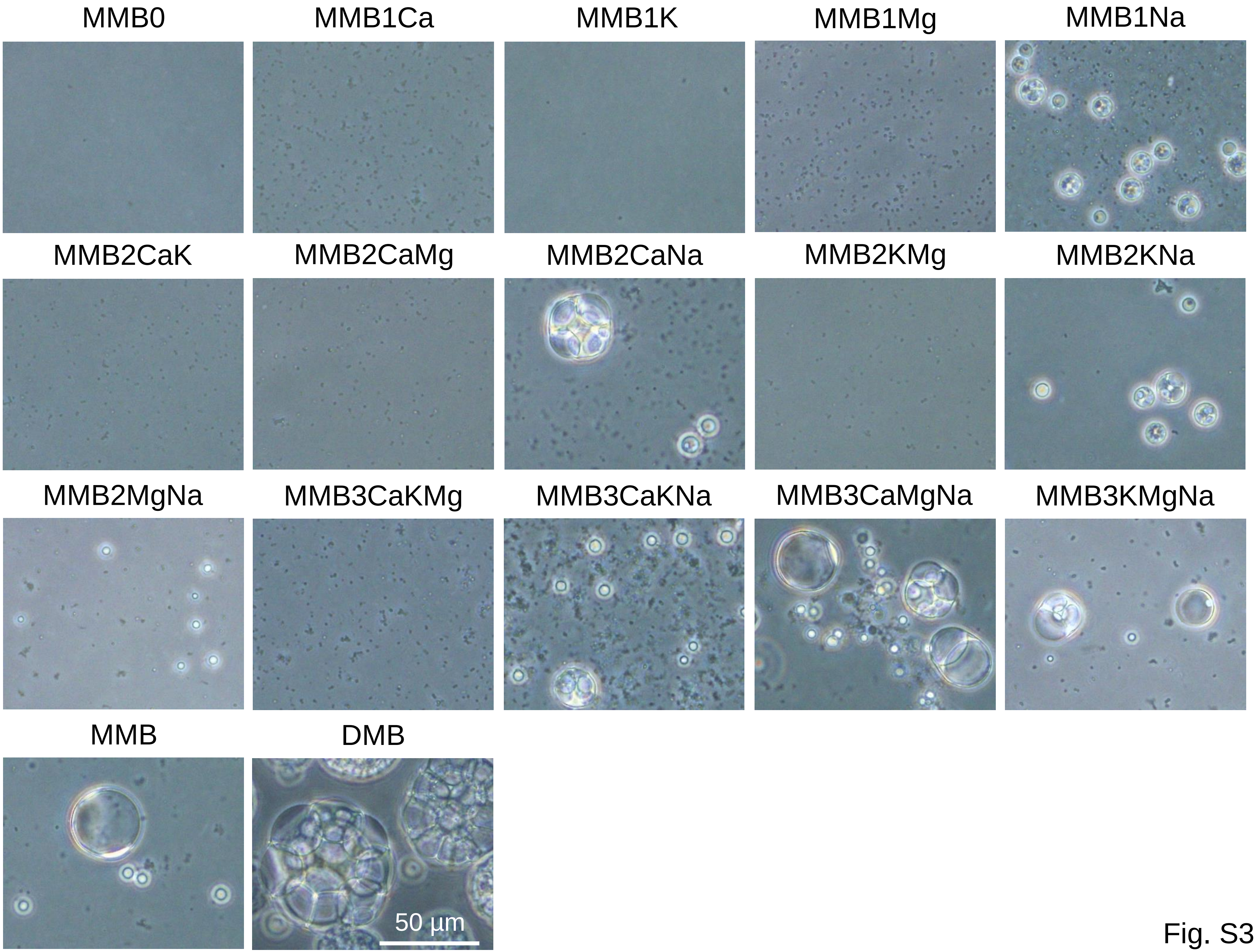
MMB0
MMB1Ca
MMB1K
MMB1Mg
MMB1Na
MMB2CaK
MMB2CaMg
MMB2CaNa
MMB2KMg
MMB2KNa
MMB2MgNa
MMB3CaKMg
MMB3CaKNa
MMB3CaMgNa
MMB3KMgNa
MMB
DMB
50 µm

Fig. S3

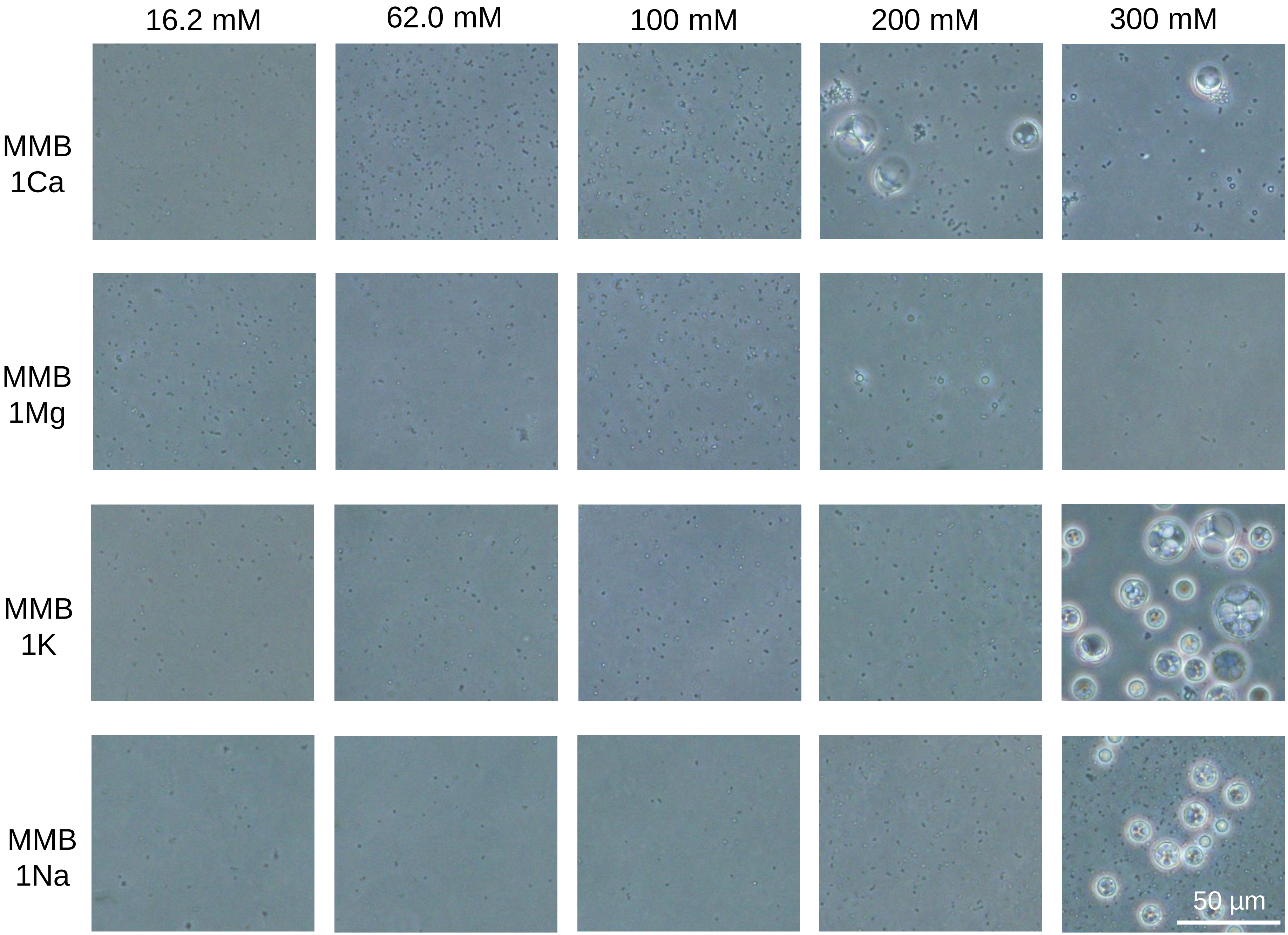
16.2 mM
62.0 mM
100 mM
200 mM
300 mM
MMB 1Ca
MMB 1Mg
MMB 1K
MMB 1Na
50 µm

Fig. S4

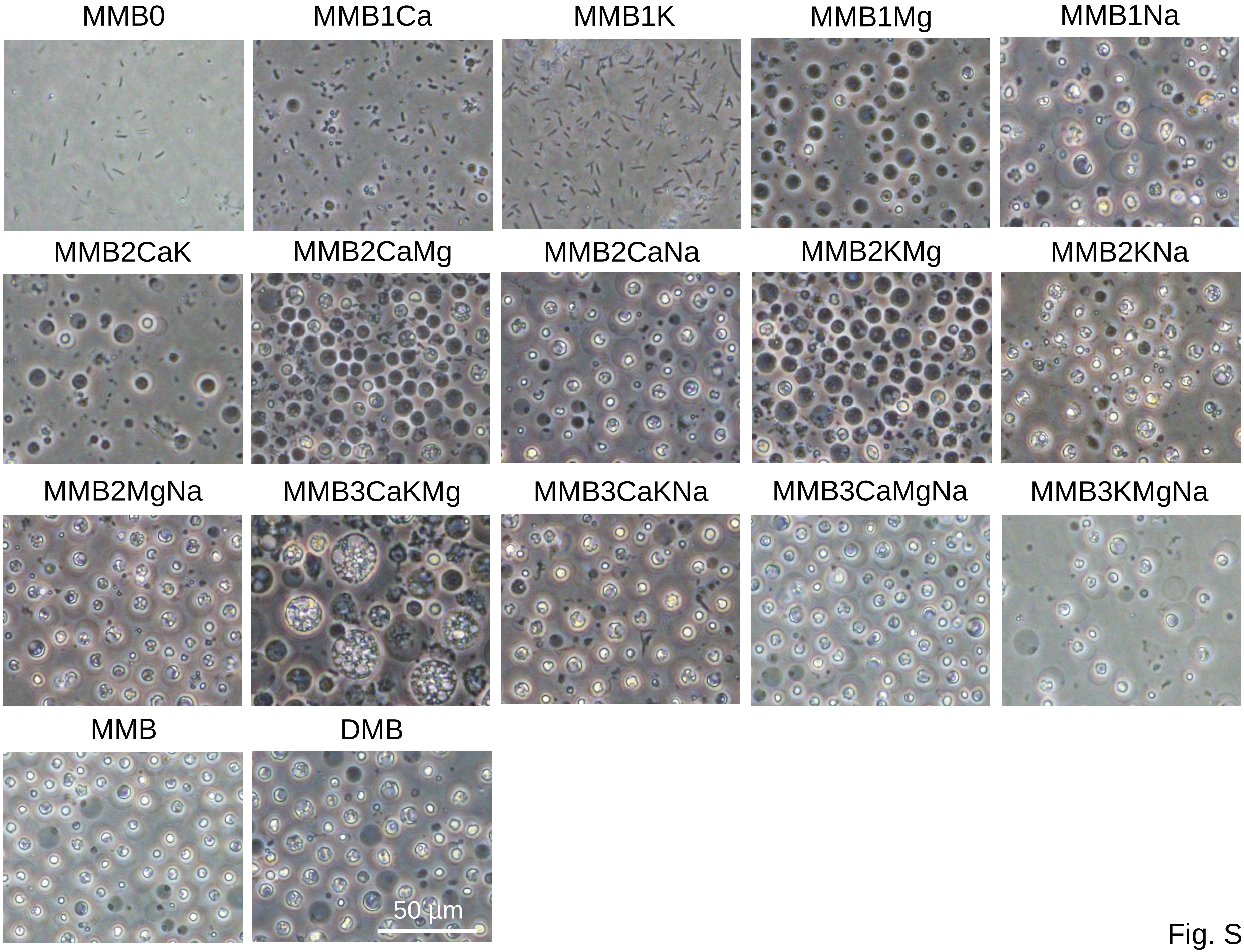
MMB0
MMB1Ca
MMB1K
MMB1Mg
MMB1Na
MMB2CaK
MMB2CaMg
MMB2CaNa
MMB2KMg
MMB2KNa
MMB2MgNa
MMB3CaKMg
MMB3CaKNa
MMB3CaMgNa
MMB3KMgNa
MMB
DMB
50 µm

Fig. S5

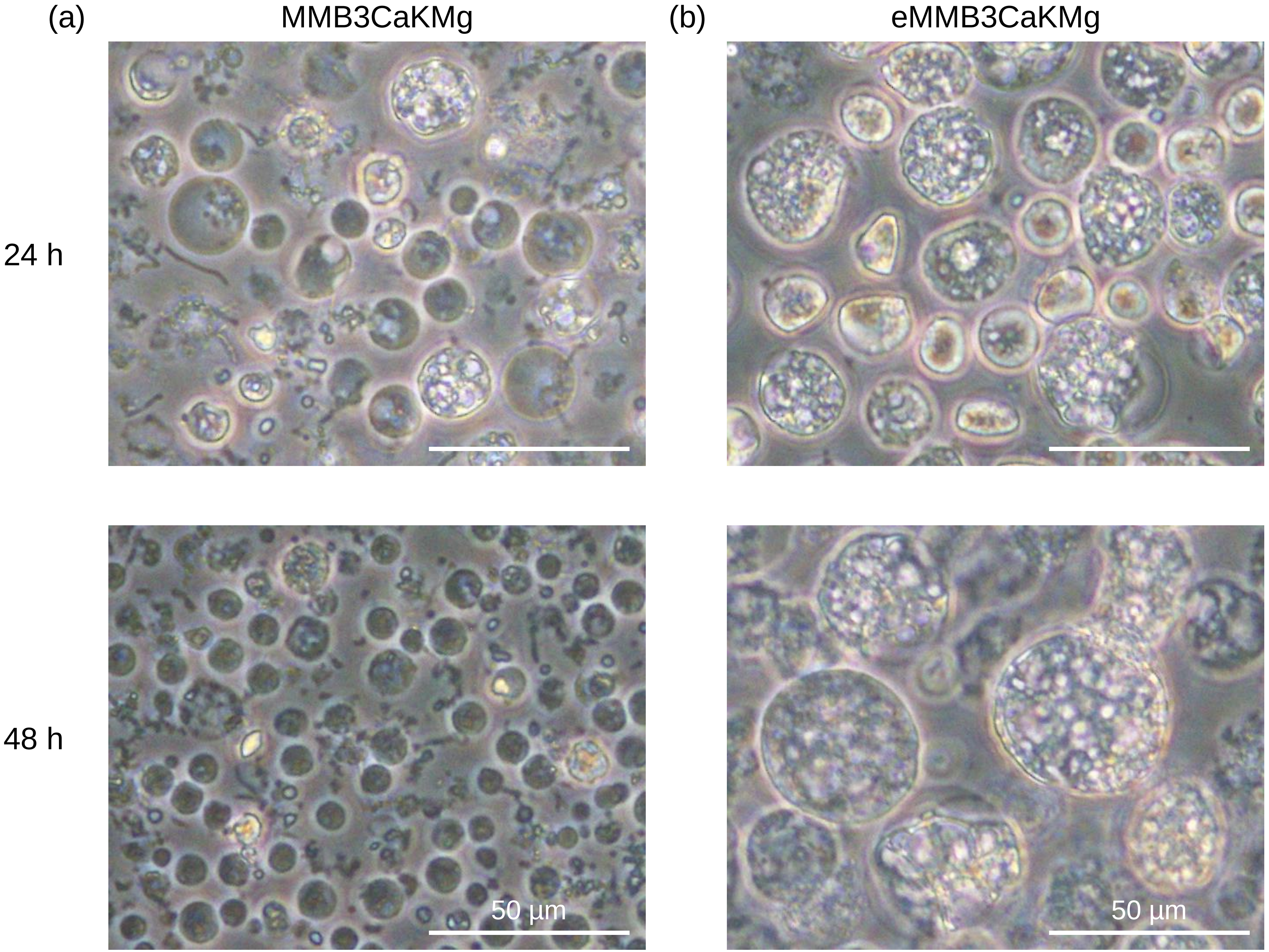

Fig. S6

(a) *E. faecalis* (b) *L. amnigena*

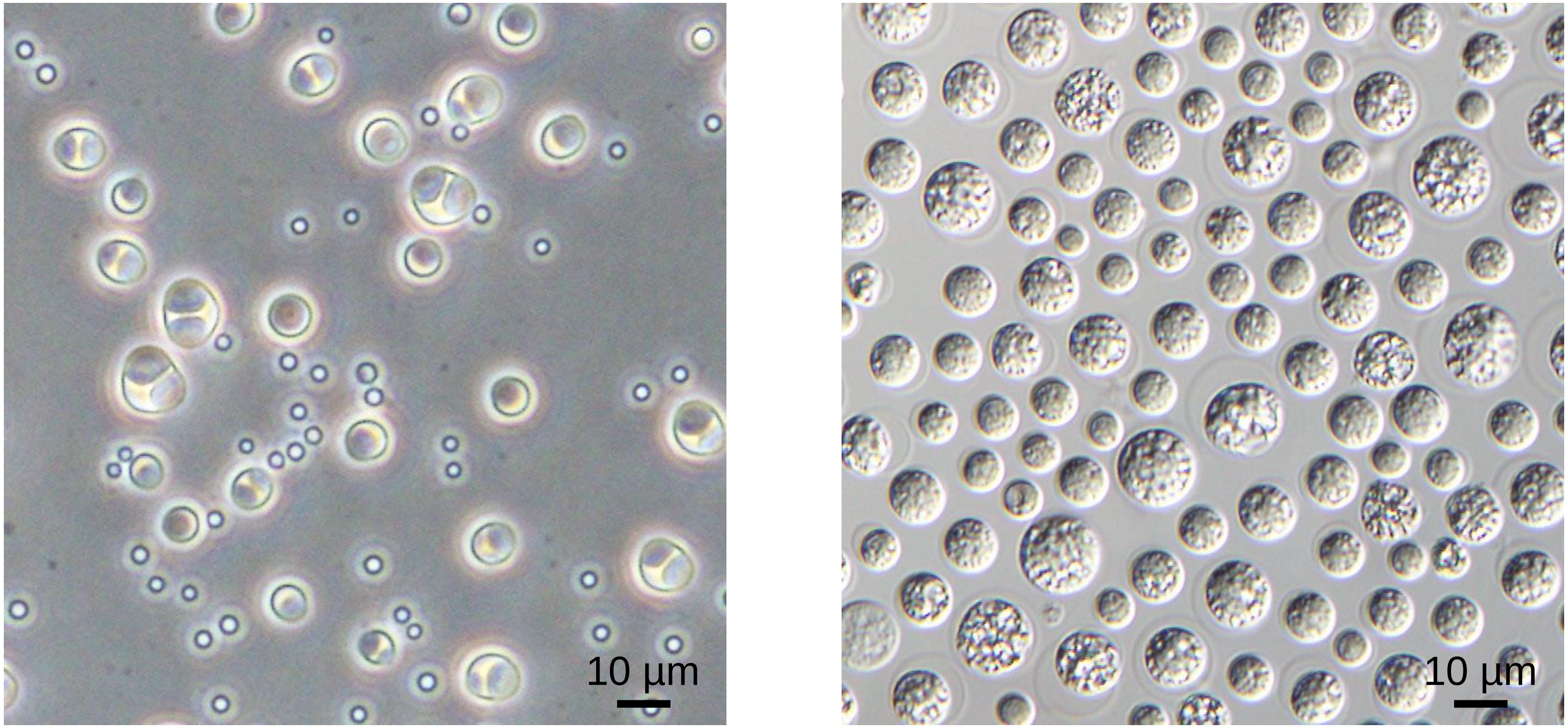

Fig. S7

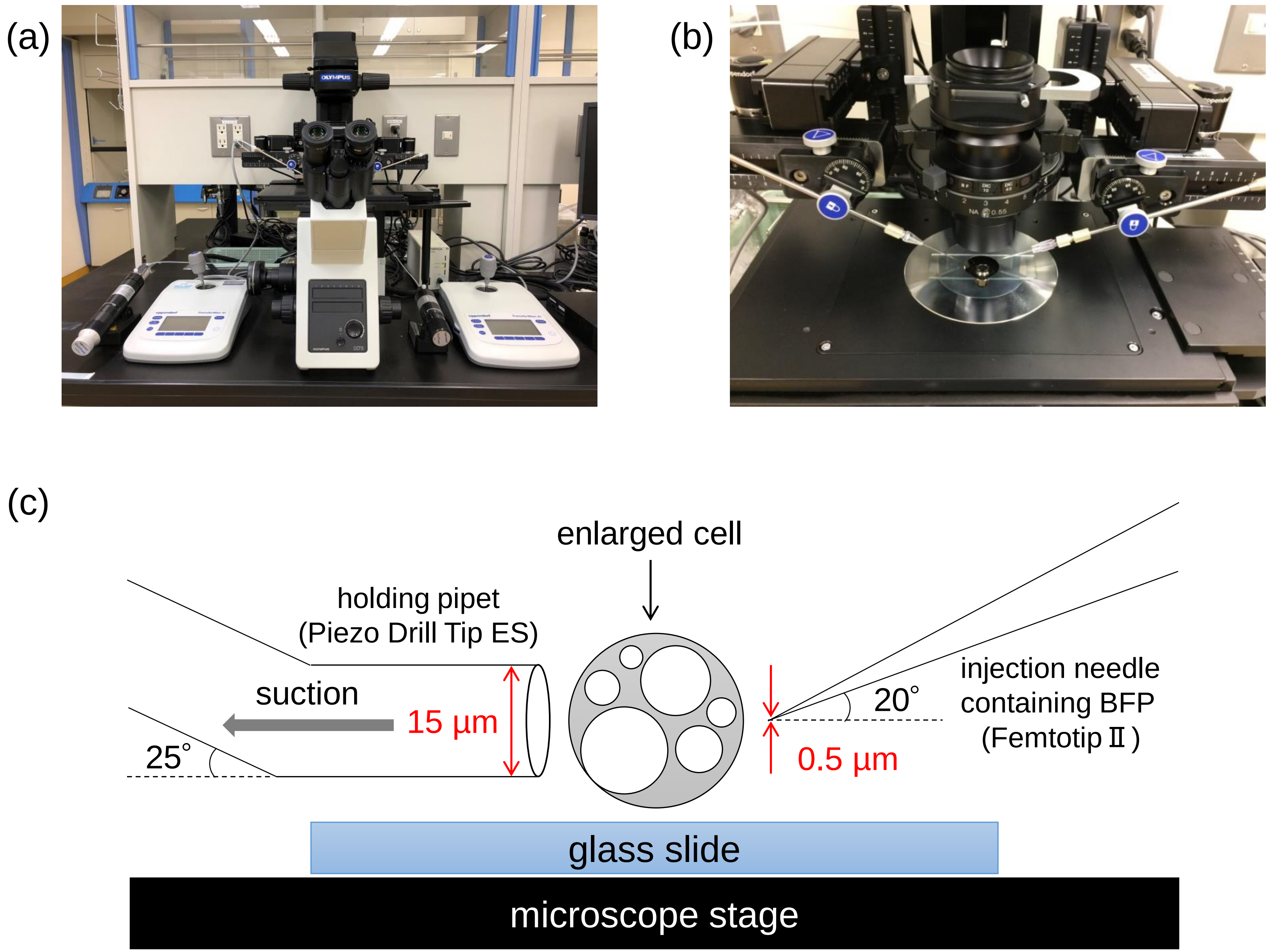
(a)
(b)
(c)
enlarged cell
holding pipet
(Piezo Drill Tip ES)
suction
15 µm
25°
20°
0.5 µm
injection needle
containing BFP
(Femtotip Ⅱ)
glass slide
microscope stage

Fig. S8

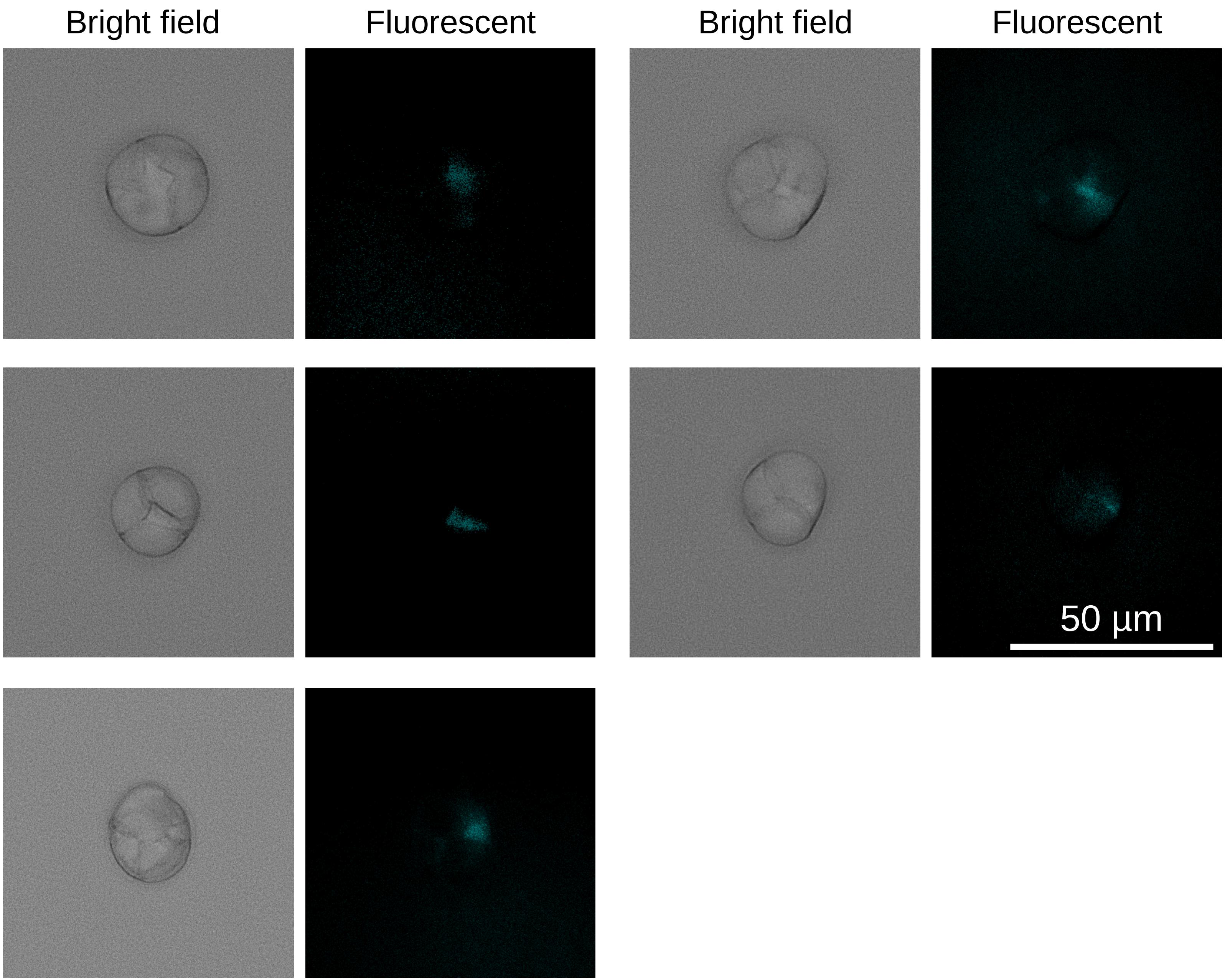

Fig. S9

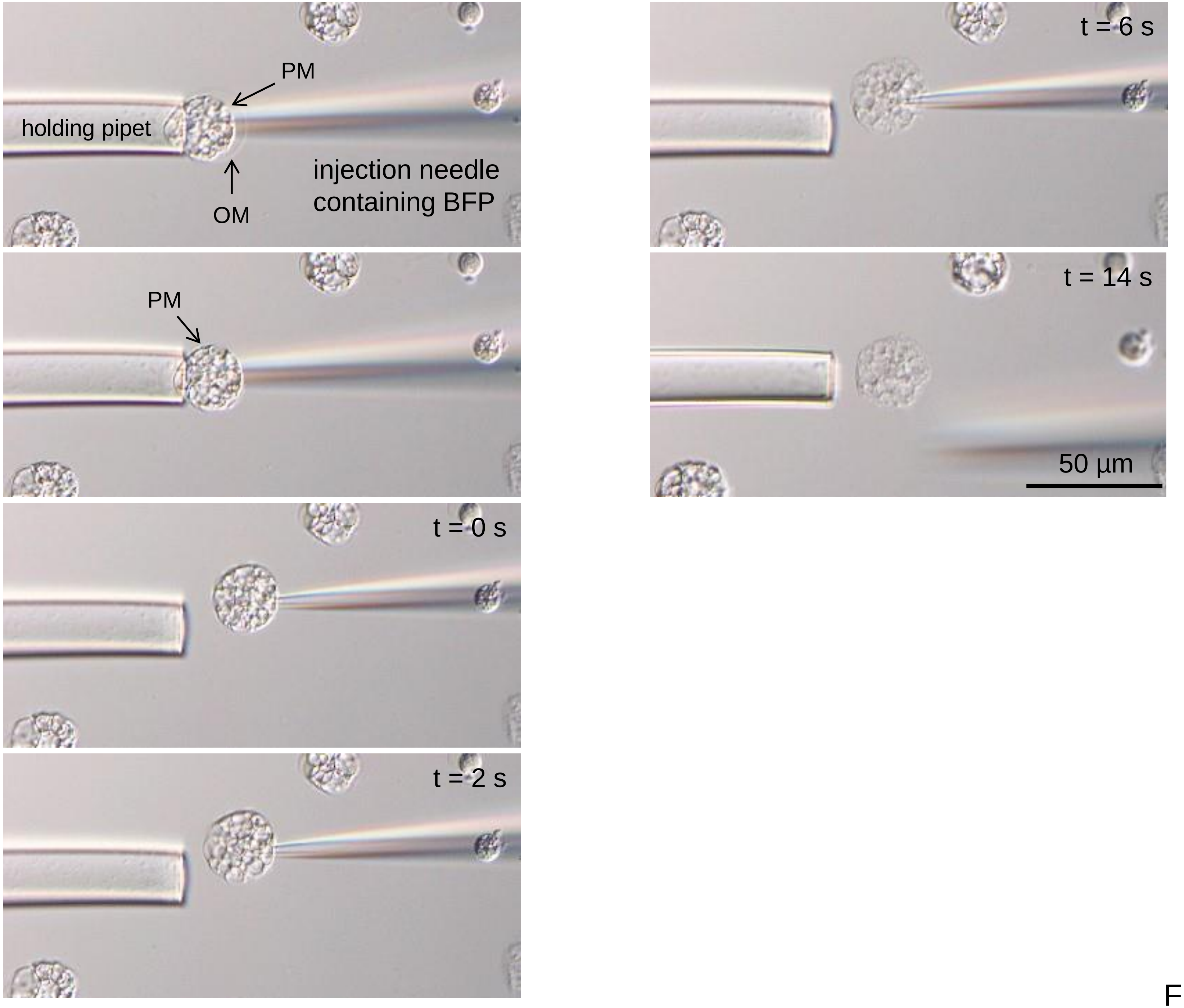
PM
holding pipet
OM
injection needle
containing BFP
PM
t = 0 s
t = 2 s
t = 6 s
t = 14 s
50 µm

Fig. S10

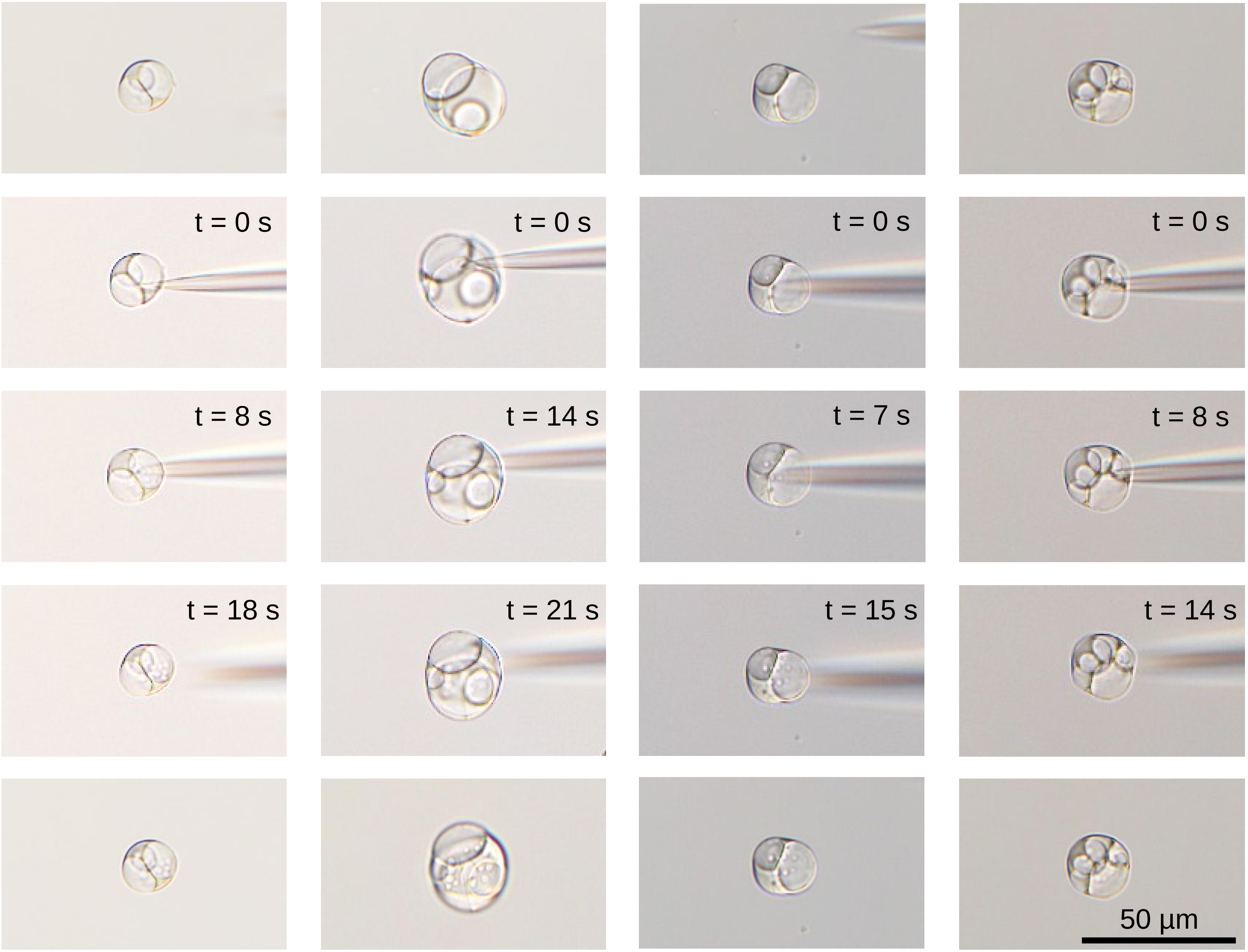

Fig. S11
